# Recent advances in the gold-catalyzed additions to C–C multiple bonds

**DOI:** 10.3762/bjoc.7.103

**Published:** 2011-07-04

**Authors:** He Huang, Yu Zhou, Hong Liu

**Affiliations:** 1 State Key Laboratory of Drug Research, Shanghai Institute of Materia Medica, Chinese Academy of Sciences, Shanghai 201203, China

**Keywords:** asymmetric addition, catalysis, gold, C−C multiple bonds, tandem reaction

## Abstract

C–O, C–N and C–C bonds are the most widespread types of bonds in nature, and are the cornerstone of most organic compounds, ranging from pharmaceuticals and agrochemicals to advanced materials and polymers. Cationic gold acts as a soft and carbophilic Lewis acid and is considered one of the most powerful activators of C–C multiple bonds. Consequently, gold-catalysis plays an important role in the development of new strategies to form these bonds in more convenient ways. In this review, we highlight recent advances in the gold-catalyzed chemistry of addition of X–H (X = O, N, C) bonds to C–C multiple bonds, tandem reactions, and asymmetric additions. This review covers gold-catalyzed organic reactions published from 2008 to the present.

## Review

### 1 Introduction

Gold-catalyzed reactions have emerged as a powerful synthetic tool in modern organic synthesis. This past decade has been the boom time for homogeneous gold catalysis, which was rather limited in organic synthesis until the advantages of gold complexes as catalysts were discovered [1]. In comparison to other transition-metal catalysts, most gold-catalyzed reactions are atom-economic, remarkably mild with regard to reaction conditions, and most importantly, have a different reaction scope [2–4].

One of the most important fundamental reactions in gold-catalyzed synthesis is the addition of X–H (X = O, N, C) bonds to C–C multiple bonds, which features diverse functional group tolerance and the easy formation of carbon–carbon and carbon–heteroatom bonds [1,4–5]. Furthermore, the rapid growing area of tandem reactions has allowed chemists to assemble diverse complex molecular frameworks more conveniently. Although various research efforts have led to gold-catalyzed addition reactions, the area of asymmetric addition has only recently been pioneered. Currently, a broad range of chiral gold catalysts (or gold combined with chiral ligands) has been developed and screened. However, only limited success has been achieved. The most notable example is the chiral BIPHEP-based catalyst, which has been successfully employed in several asymmetric cycloadditions.

Several early reviews have summarized well the progress of gold-catalyzed reactions up to 2008 [6–16]. Since then, the expansion of this field has continued unabated as evidenced by more than 500 publications to be found in the literature. Herein, we summarize the new research efforts that cover several aspects of gold-catalyzed additions to unsaturated bonds: (i) X–H (X = O, N, C) bonds to C–C multiple bonds; (ii) tandem reactions; and (iii) gold-catalyzed asymmetric additions. The literature published from 2008 up to the February of 2011 is covered. Only the most important recent studies have been selected to demonstrate the significance of gold catalysis.

### 2 Gold-catalyzed C–O bond formations

The carbon–oxygen bond is one of the most widespread types of bonds in nature. Gold catalytic addition of oxygen nucleophiles to electronically non-activated C–C multiple bonds represents an attractive approach to the synthesis of functionalized ethers and ketones. In particular, the intramolecular addition of oxygen nucleophile to C–C multiple bonds has become a very effective tool in the synthesis of oxygen heterocycles from readily available starting materials [11].

#### 2.1 Alcohols, phenols and epoxides as nucleophiles

**Scheme 1:**
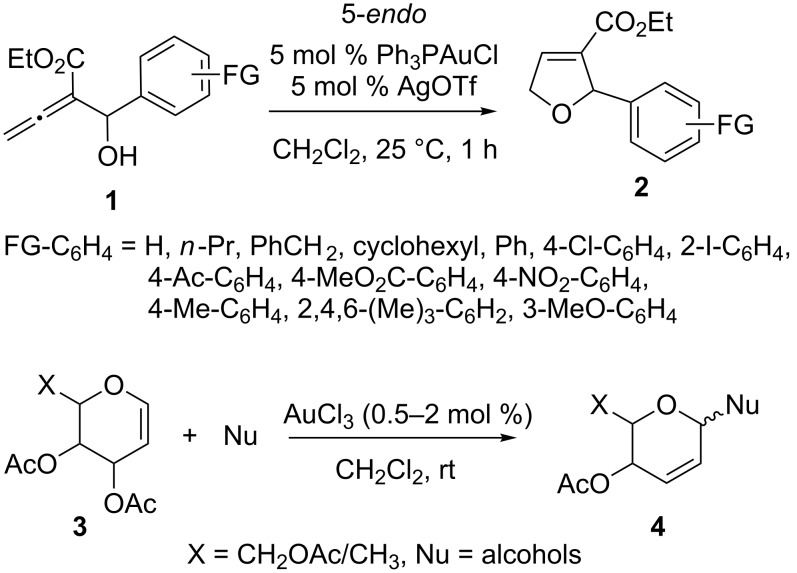
Gold-catalyzed addition of alcohols.

In general, dihydrofuran analogs can be constructed from alkynes by palladium-catalyzed intramolecular hydroalkoxylation reactions. However, the more common way to synthesize dihydrofurans is the gold catalyzed cyclization of vinyl allenols [17]. For instance, hydroxyallenic esters **1** can be selectively transformed into 2-alkyl- and 2-aryl-3-ethoxycarbonyl-2,5-dihydrofurans **2** by Ph_3_PAuCl and AgOTf through intramolecular hydroalkoxylation via a 5-*endo* mode [18]. Gold(III) chloride in catalytic amounts activates 3,4,6-tri-*O*-acetyl-D-glucal, 3,4,6-tri-*O*-acetyl-D-galactal, and 3,4-di-*O*-acetyl-L-rhamnal **3** efficiently. The activated species can be employed in the Ferrier reaction with different nucleophiles at ambient conditions to yield the unsaturated derivatives **4** (Scheme 1) [19].

**Scheme 2:**
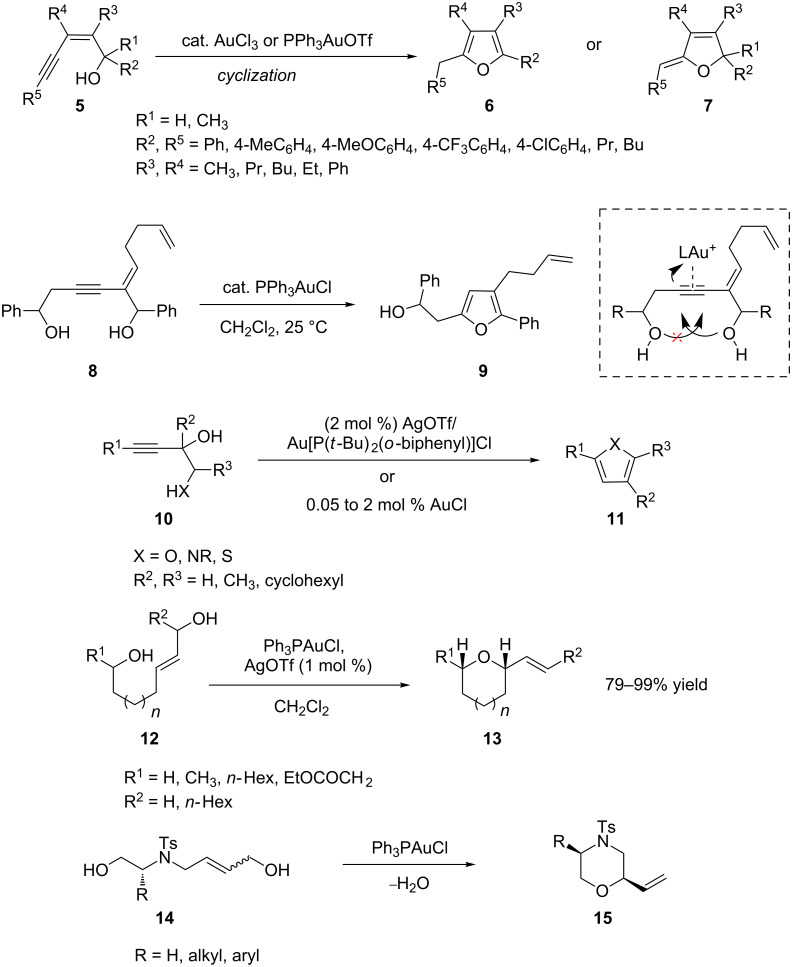
Gold-catalyzed cycloaddition of alcohols.

The intramolecular addition of a hydroxy group to a carbon–carbon triple bond is an effective strategy to construct furan analogues. Du et al. reported a highly efficient Au-catalyzed cyclization of (*Z*)-enynols that proceeded under mild reaction conditions. This methodology provided rapid access to substituted furans **6** and stereo-defined (*Z*)-5-ylidene-2,5-dihydrofurans **7** in a regioselective manner from suitably substituted (*Z*)-2-en-4-yn-1-ols **5** [20]. A similar strategy has been applied to an efficient formation of substituted furans **9** through gold-catalyzed selective cyclization of enyne-1,6-diols **8** [21]. Nucleophilic attack of the hydroxy oxygen atom on 1-position to a gold-coordinated C–C triple bond formed the vinyl–gold complex. Surprisingly, no other cyclic compound formed by nucleophilic attack of the hydroxy oxygen atom on C-6-position to a gold-coordinated C–C triple bond was formed. A new efficient route to furans **11** by gold-catalyzed intramolecular nucleophilic attack of readily available heteroatom-substituted propargyl alcohols **10** has been developed by Aponick and co-workers [22]. For the formation of tetrahydropyran analogs **13** and **15**, the gold(I)-catalyzed cyclization of monoallylic diols **12** and **14** is an efficient method (Scheme 2) [23–24].

**Scheme 3:**
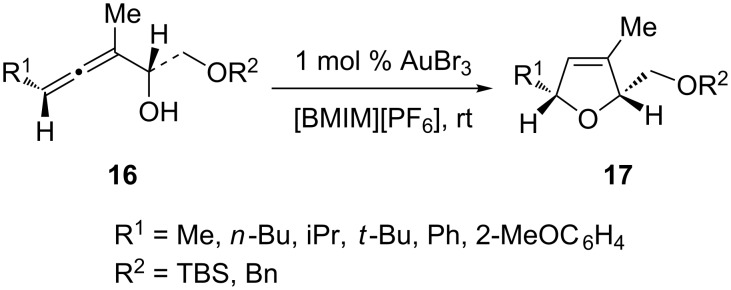
Ionic liquids as the solvent in gold-catalyzed cycloaddition.

In addition to common organic solvents, an attractive alternative is the use of ionic liquids as the reaction solvent, which often affords inexpensive, recyclable (and therefore environmentally benign), and sustainable catalyst systems. For example, Aksin et al. demonstrated that ionic liquids were highly suitable reaction media for the gold-catalyzed cycloisomerization of α-hydroxyallenes **16** to 2,5-dihydrofurans **17** (Scheme 3) [25]. The best system was found to be AuBr_3_ in [BMIM][PF_6_]. The cycloisomerization of various alkyl- or arylsubstituted α-hydroxyallenes gave corresponding 2,5-dihydrofuran with complete axis-to-center chirality transfer.

**Scheme 4:**
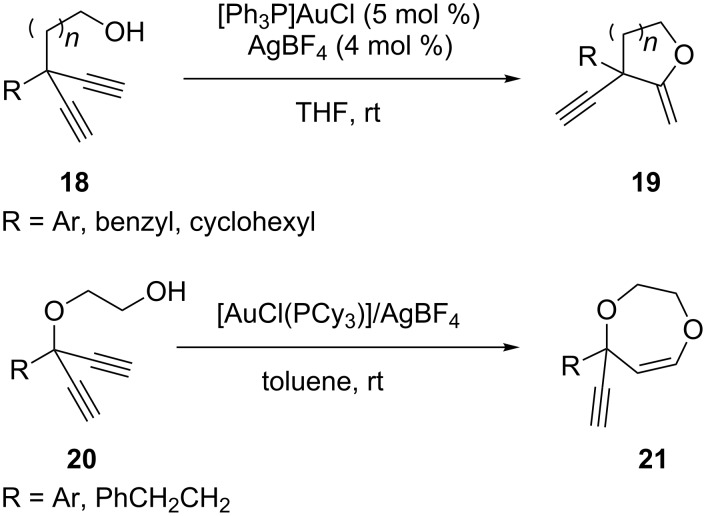
Gold-catalyzed cycloaddition of diynes.

Rüttinger et al. reported a gold-catalyzed synthetic route for the preparation of enynes (Scheme 4) [26]. The gold-catalyzed cyclization provided the corresponding *exo*-enol ethers **19** in moderate to high yield with complete regioselectivity. By contrast, Wilckens et al. reported the gold-catalyzed *endo-*cyclizations of 1,4-diynes **20** to seven-membered ring heterocycles **21** [27]. The cyclization occurs exclusively in an *endo-*fashion under mild conditions and provides access to dihydrodioxepines and tetrahydrooxazepines.

**Scheme 5:**
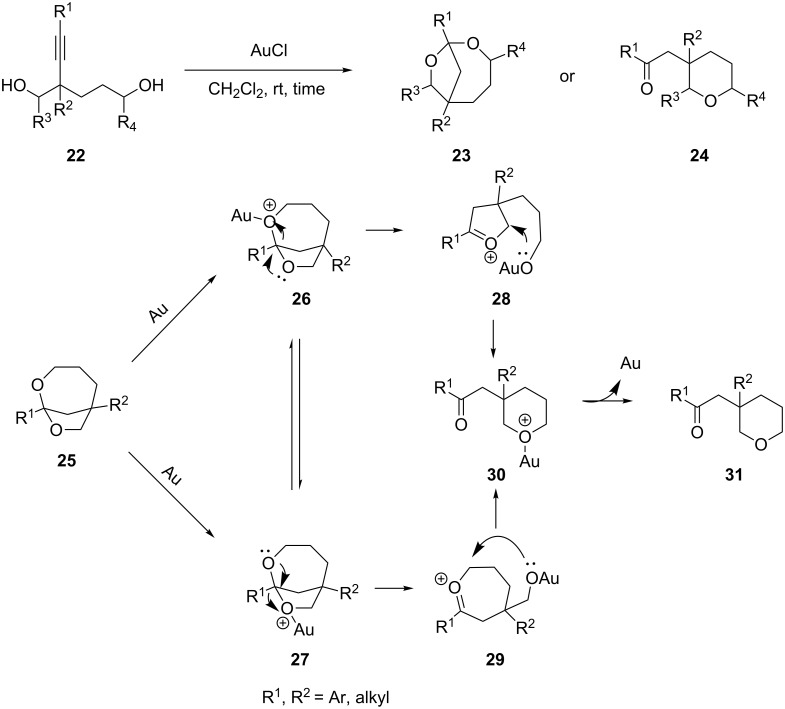
Gold(I) chloride catalyzed cycloisomerization of 2-alkynyl-1,5-diols.

The dioxabicyclo[4.2.1] ketal **23** and its further transformation product tetrahydropyran **24** were produced by an efficient gold(I) chloride catalyzed cycloisomerization of 2-alkynyl-1,5-diol **22** [28]. A plausible mechanism for the gold-catalyzed transformation of dioxabicyclo[4.2.1]ketal **25** to tetrahydropyran **31** is outlined in Scheme 5. The gold catalyst activates one of the oxygen atoms to form the intermediates **26** or **27**, which then rearrange to yield the oxonium intermediates **28** or **29**, respectively.

**Scheme 6:**
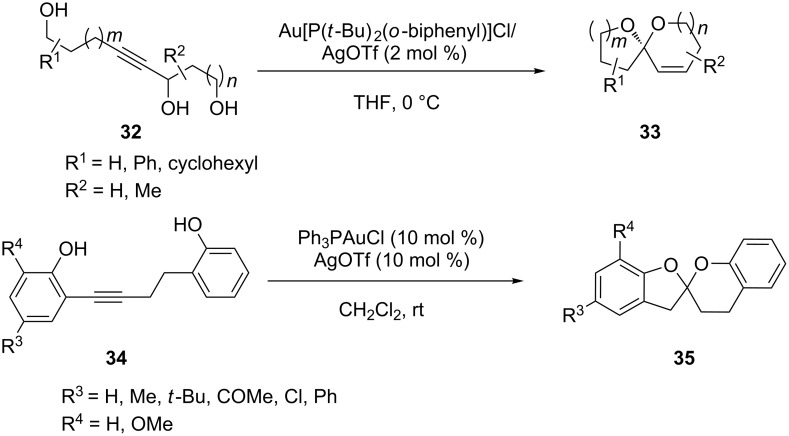
Gold-catalyzed cycloaddition of glycols and dihydroxy compounds.

Gold(I)-catalyzed intramolecular cyclization of monopropargylic triols **32** has been reported to be a novel and mild approach [29] for producing olefin-containing spiroketals **33** (and enantiomer) in excellent yields (Scheme 6). A range of variously substituted triols was prepared which were cyclized to give substituted 5- and 6-membered ring spiroketals. Similarly, the synthesis of the bisbenz-annelated spiroketal core **35** of natural bioactive rubromycins via a gold-catalyzed double intramolecular hydroalkoxylation was reported by Zhang and co-workers [30]. A tandem cyclization mechanism was proposed by the authors.

**Scheme 7:**
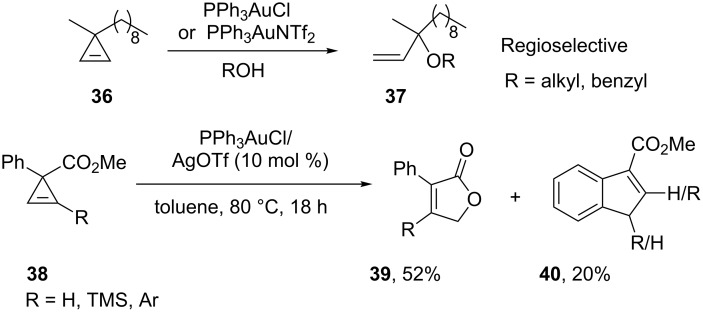
Gold-catalyzed ring-opening of cyclopropenes.

**Scheme 8:**
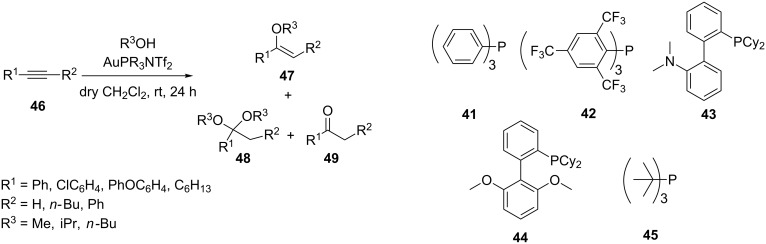
Gold-catalyzed intermolecular hydroalkoxylation of alkynes. PR_3_ = **41–45**.

The first example of gold-catalyzed ring-opening addition of cyclopropenes has been developed by Lee’s group [31–32]. The reaction of alkyl-disubstituted cyclopropene **36** with a series of alcohols generated the corresponding *tert*-allylic ethers **37** with high regioselectivity. Gold(I) catalysts were found to be unique and superior in terms of reactivity and regioselectivity. A notable observation in some of these studies is that gold(I) catalyzed rearrangement to furanones **39** and indenes **40** is observed upon introduction of ester and phenyl substituents on the cyclopropene (Scheme 7). AuPR_3_NTf_2_ complexes (PR_3_ = **41–45**) are selective catalysts for the intermolecular hydroalkoxylation of electron-poor alkynes of type R−C≡C−EWG and dimethyl acetylenedicarboxylate [33]. In reactions of phenylacetylene the ratio of vinyl ether **47** to ketal **48** can be controlled by the choice of catalyst (Scheme 8).

**Scheme 9:**
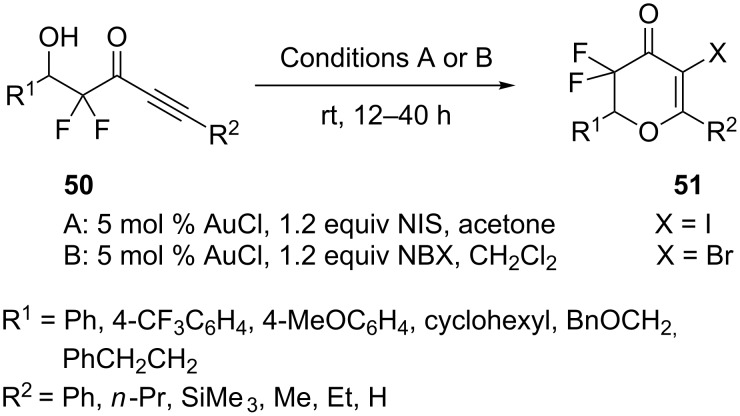
Gold-catalyzed intramolecular 6-endo-dig cyclization of β-hydroxy-α,α-difluoroynones.

**Scheme 10:**
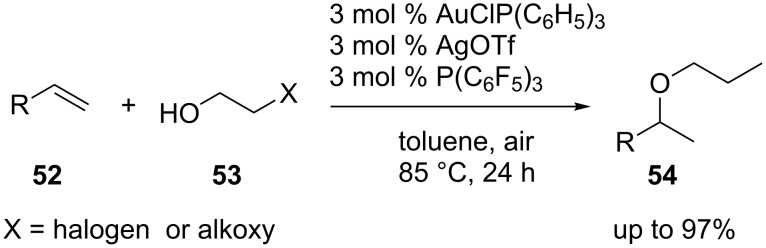
Gold-catalyzed intermolecular hydroalkoxylation of non-activated olefins.

The gold-catalyzed intramolecular 6-endo-dig cyclization of β-hydroxy-α,α-difluoroynones **50** under mild conditions has been developed (Scheme 9) [34]. The result indicated that gold catalysis is compatible with electrophilic fluorinating reagents. Furthermore, it is possible to couple the 6-endo-dig cyclization with iodination and bromination of the presumed vinyl–gold intermediate. However, attempted alkoxychlorination with *N*-chlorosuccinimide failed. Intermolecular hydroalkoxylation of non-activated olefins catalyzed by the combination of gold(I) and electron deficient phosphine ligands has been developed [35]. Gold-catalyzed hydroalkoxylations of non-activated olefins **52** and simple aliphatic alcohols **53** gave unsatisfactory results. However, a significant improvement of reaction efficiency was observed by employing alcohol substrates bearing coordination functionalities. In addition, the catalyst system with electron deficient phosphines was also found to catalyze the desired reaction effectively (Scheme 10).

**Scheme 11:**
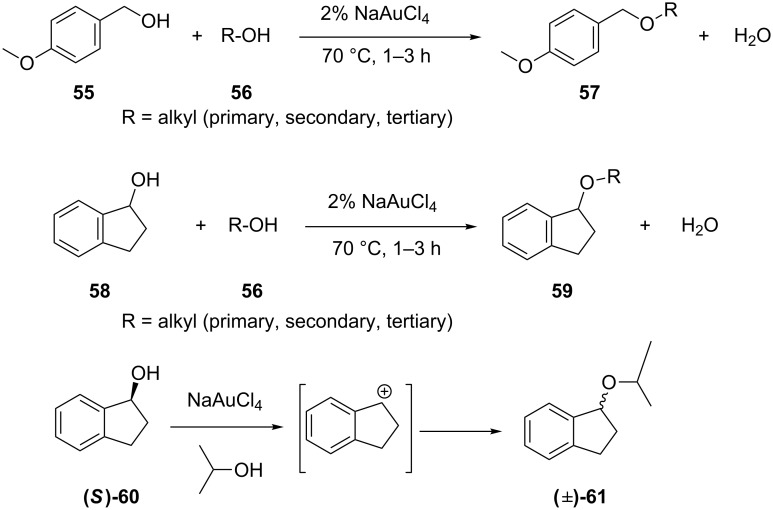
Preparation of unsymmetrical ethers from alcohols.

An efficient approach [36] for the preparation of unsymmetrical ethers from alcohols has been developed by utilizing NaAuCl_4_. The benzylic and secondary alcohols (**55** and **58**) worked well under mild conditions with low catalyst loading (Scheme 11). The chiral benzyl alcohol **60** gave racemic ether **61**, which suggested the intermediacy of a carbocation.

**Scheme 12:**
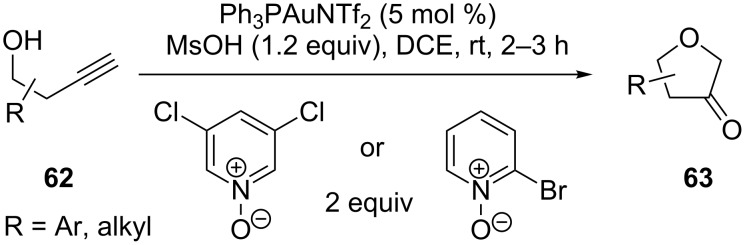
Expedient synthesis of dihydrofuran-3-ones.

Ye et al. reported an expedient gold-catalyzed synthesis of dihydrofuran-3-ones **63**, in which terminal alkynes **62** were used as equivalents of α-diazo ketones to generate α-oxo gold carbenes (Scheme 12) [37]. The α-oxo gold carbenes were produced via gold-catalyzed intermolecular oxidation of **62**. This provides improved synthetic flexibility in comparison with the intramolecular strategy and offers a safe and economical alternative to those based on diazo substrates.

**Scheme 13:**
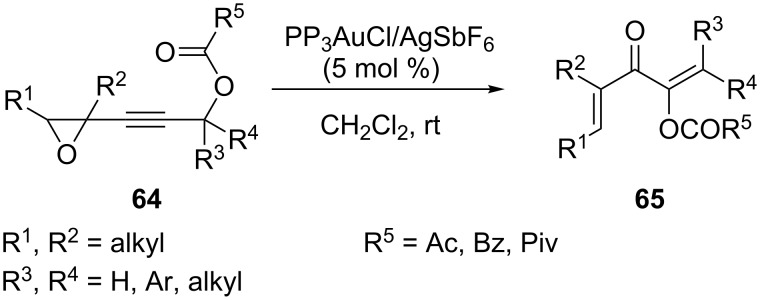
Catalytic approach to functionalized divinyl ketones.

A catalytic approach to functionalized divinyl ketones through a gold-catalyzed rearrangement of (3-acyloxyprop-1-ynyl)oxiranes **64** has also been developed [38]. The reaction proceeds via rearrangement of (3-acyloxyprop-1-ynyl)oxiranes to acyloxydivinyl ketones, migration of the adjacent acyloxy group, as well as cycloreversion of oxetene and provides easy access to a variety of acyloxyl divinyl ketones **65** (Scheme 13).

**Scheme 14:**
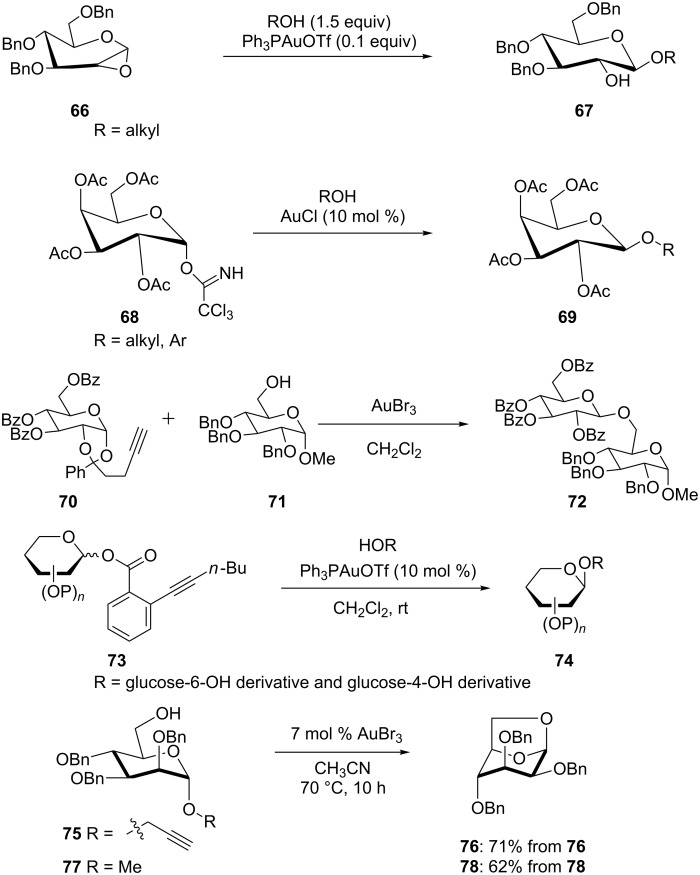
Gold-catalyzed glycosylation.

A number of interesting gold-catalyzed glycosylations have appeared in recent years. Ph_3_PAuOTf is reported to be a superior catalyst (yield increases by >20%) compared to conventionally used ZnCl_2_ for the well-established glycosylation reaction with 1,2-anhydrosugars **66** as donors (Scheme 14) [39]. The gold(I)-catalyzed reaction of 2,3,4,6-tetra-*O*-acetyl-α-D-galactopyranosyl trichloroacetimidate (**68**) with alcohols gave β-galactosides **69** stereoselectively and in much higher yields compared to those obtained with 2,3,4,6-tetra-*O*-acetyl-α-D-galactopyranosyl bromide [40]. Subsequently, a method to activate the propargyl 1,2-orthoesters **70** selectively in the presence of propargyl glycosides and propargyl ethers was developed [41]. Recently, Li et al. reported the gold(I)-catalyzed glycosylation with glycosyl *ortho*-alkynylbenzoates **73** as donors [42]. This glycosylation protocol was used in an efficient synthesis of a cyclic triterpene tetrasaccharide **74**, which demonstrated its versatility and efficacy. Another study [43] showed that 1,6-anhydro sugars **76** and **78** could be synthesized by utilizing salient features of gold-catalyzed glycosidations.

#### 2.2 Aldehydes and ketones as nucleophiles

**Scheme 15:**
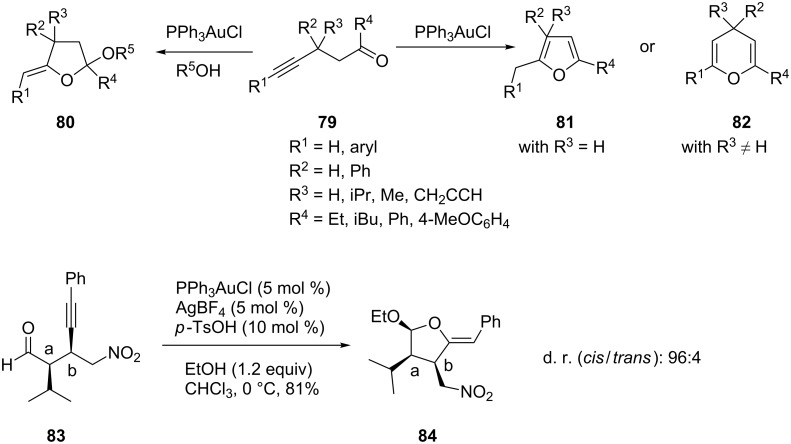
Gold-catalyzed cycloaddition of aldehydes and ketones.

Different oxygen heterocycles can be obtained from the gold-catalyzed cyclization of alk-4-yn-1-ones **79** depending on the substitution pattern in the substrate and the reaction solvent. Thus, alkynones with one substituent at C-3 undergo a *5*-exo-dig cycloisomerization to yield substituted furans **81**, whilst substrates bearing two substituents at C-3 undergo a *6*-endo-dig cyclization to give 4*H*-pyrans **82**. By contrast, alkylidene/benzylidene-substituted tetrahydrofuranyl ethers **80** are formed in a tandem nucleophilic addition/cycloisomerization in alcoholic solvents [44]. Similarly, Belot et al. reported a gold-catalyzed cyclization which led to nitro-substituted tetrahydrofuranyl ethers **84** (Scheme 15) [45].

**Scheme 16:**
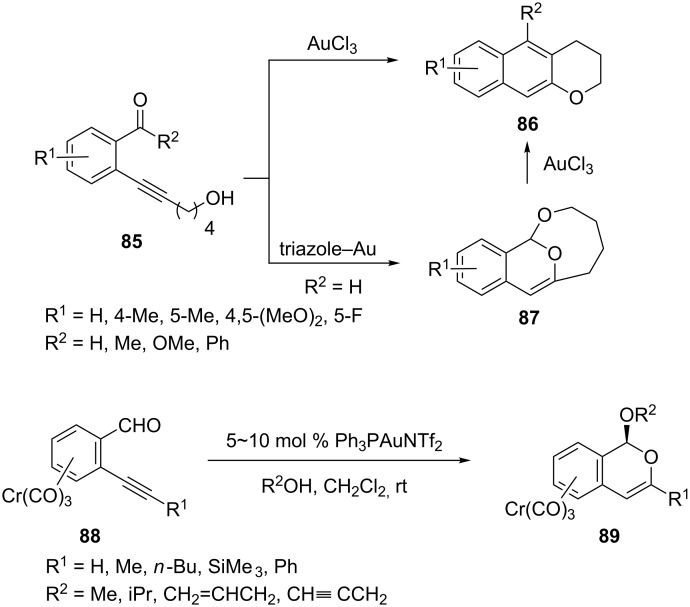
Gold-catalyzed annulations of 2-(ynol)aryl aldehydes and *o*-alkynyl benzaldehydes.

Liu et al. have developed a facile synthesis of benzochromanes **86** and benzobicycloacetals **87** from the gold-catalyzed cascade annulations of 2-(ynol)aryl aldehydes **85** [46]. Benzochromanes were obtained when AuCl_3_ was employed as the catalyst, whereas benzobicyclo[5.3.1]acetals **87** were produced when triazole–gold was employed as the catalyst. With alcohol nucleophiles, gold(I)-catalyzed cyclization of *o*-alkynyl benzaldehyde **88** and benzaldimine–chromium complexes gave stereoselectively 1-anti-functionalized heterocycle chromium complexes **89** (Scheme 16) [47]. This made the methodology useful for the synthesis of enantiomerically pure *trans-* and *cis*-1,3-dimethylisochromans starting from a single planar chiral chromium complex.

#### 2.3 Carboxylates as nucleophiles

**Scheme 17:**
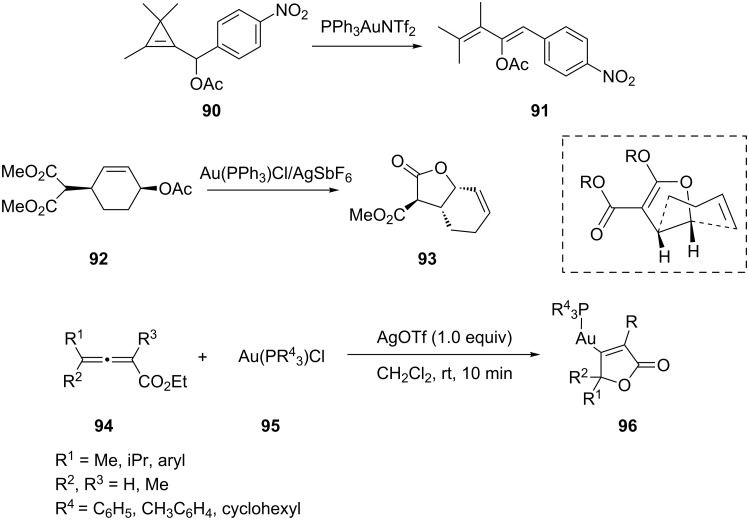
Gold-catalyzed addition of carboxylates.

Seraya has reported the gold-catalyzed rearrangement of cyclopropenylmethyl acetates as a route to (*Z*)-acetoxydienes [48]. Thus, treatment of 4-nitrobenzaldehyde derived cyclopropene **90** with a catalytic amount of PPh_3_AuNTf_2_ in DCM led to quantitative formation of acetoxy diene **91** with a 4:1 *Z*:*E* selectivity within 5 min at −50 °C. Wang et al. developed an efficient method for the preparation of polysubstituted C–vinyl butyrolactones through a gold-catalyzed highly diastereoselective cyclization of malonate substituted allylic acetates [49]. As an example, treatment of *syn*-4-acetoxycyclohexenyl malonate **92** with a catalytic amount of AuPPh_3_Cl/AgSbF_6_ in DCE at 70 °C for 3 h led to the isolation of 3,4-*anti*-4,5-*syn*-3-methoxycarbonyltetrahydrobenzobutyrolactone **93** in 80% yield. The possible intermediate is shown in Scheme 17. Using the AuPPh_3_Cl/AgOTf system as the equivalent of AuPPh_3_OTf, Liu et al. found that the in situ generated cationic Au(I) reagent reacted with ethyl α-methyl-γ-cyclohexyl allenoate in dichloromethane at room temperature to form the gold complex **96** in 85% yield (Scheme 17) [50]. This result could provide the experimental evidence required to support the postulated mechanism of Au-catalyzed reactions.

**Scheme 18:**
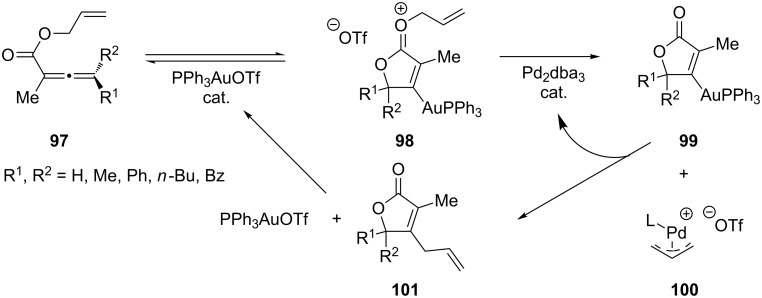
Dual-catalyzed rearrangement reaction of allenoates.

Dual-catalyzed rearrangement reactions have been reported by Shi and co-workers for the preparation of substituted butenolides **101** and isocoumarins [51]. In this study, the authors employed a carbophilic Lewis acidic Au(I) catalyst to catalyze the cross-coupling reactivity of a second Lewis basic Pd catalyst in order to functionalize vinyl–gold intermediates arising from intramolecular substrate rearrangements (Scheme 18).

#### 2.4 Propargylic alcohols and propargylic carboxylate rearrangements

**Scheme 19:**
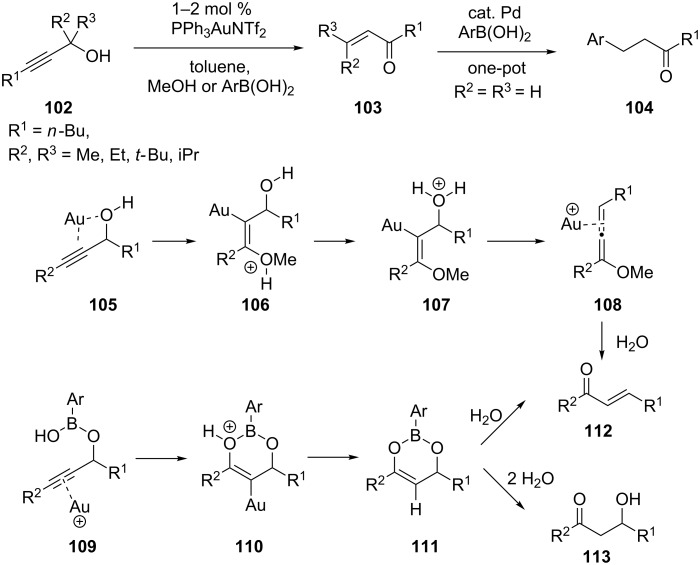
Meyer–Schuster rearrangement of propargylic alcohols.

Pennell et al. reported Meyer–Schuster rearrangements of propargylic alcohols **102** at room temperature in toluene with 1–2 mol % PPh_3_AuNTf_2_, in the presence of 0.2 equiv of 4-methoxyphenylboronic acid or 1 equiv of methanol [52]. Mechanistically, it was proposed that the enones **103** were produced through two pathways (Scheme 19).

**Scheme 20:**
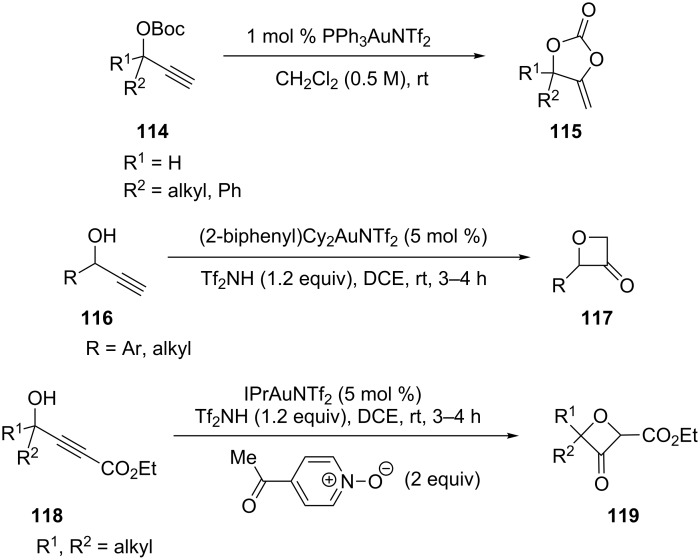
Propargylic alcohol rearrangements.

The gold(I)-catalyzed rearrangement of propargylic *tert*-butyl carbonates gave diversely substituted 4-alkylidene-1,3-dioxolan-2-ones **115** [53]. For example, treatment of propargylic *tert*-butyl carbonate **114** with 1 mol % PPh_3_AuNTf_2_ in CH_2_Cl_2_ at room temperature led to isolation of the cyclic carbonate in 83% yield. Syntheses of oxetan-3-ones typically demand multiple synthetic steps and/or highly functionalized substrates. Alternatively, Ye et al. [54] developed a practical gold-catalyzed one-step synthesis of oxetan-3-ones **117** and **119** from readily available propargylic alcohols **116** and **118**. Since chiral propargylic alcohols are readily available, this methodology provides easy access to chiral oxetan-3-ones. For example, the reaction of enantiomerically enriched secondary propargyl alcohols led to the chiral oxetan-3-one with no apparent racemization (Scheme 20).

### 3 Gold-catalyzed C–N bond formations

Many organic compounds containing nitrogen exhibit important biological and pharmaceutical properties. As with gold-catalyzed C–O bond formation, the directly catalytic addition of a nitrogen nucleophile to a C–C multiple bond represents an attractive approach to the formation of C–N bonds [55]. This is a direct and efficient procedure for the synthesis of nitrogen containing compounds of industrial importance.

#### 3.1 Alkyl- and aromatic amines as nucleophiles

**Scheme 21:**
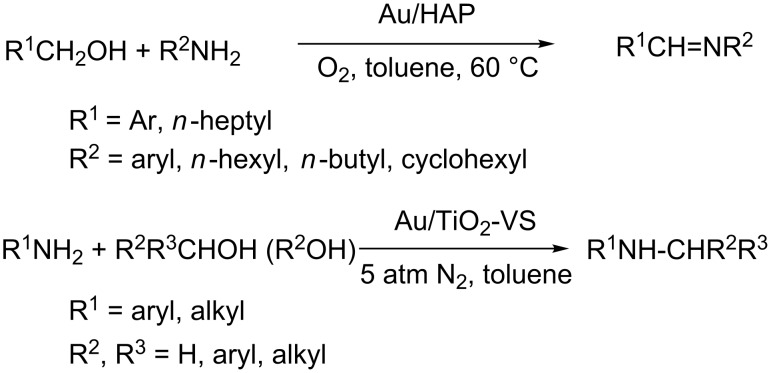
Gold-catalyzed synthesis of imines and amine alkylation.

Imines and oximes are versatile synthetic intermediates for the preparation of dyes, pharmaceuticals, and agricultural chemicals. Sun et al. have reported a multi-task Au/hydroxyapatite reagent for the heterogeneous catalyzed oxidation of alcohols and amines to imines or oximes [56]. *N*-alkylation of primary amines is an important reaction in organic synthesis. He et al. developed an efficient gold-catalyzed one-pot selective *N*-alkylation of amines with alcohols [57]. In their study, gold nanoparticles supported on titania act as an efficient heterogeneous catalyst for the reaction to give the *N*-alkylated amines in excellent yields (Scheme 21).

**Scheme 22:**
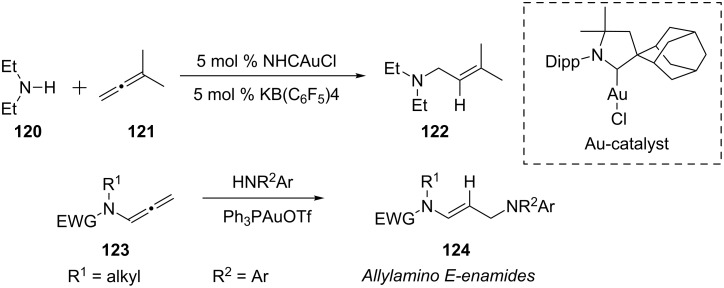
Hydroamination of allenes and allenamides.

Zeng and co-workers reported that cationic gold(I) complexes promote the addition of all types of non-tertiary amines **120** to a variety of allenes **121** to afford allylic amines **122** in good to excellent yields [58]. Importantly, the Markovnikov adduct was obtained in all cases. A similar Markovnikov hydroamination [59] could also be achieved via an intermolecular hydroamination of allenamides **123** with arylamines under mild AuPPh_3_OTf catalysis conditions to furnish allylamino (*E*)-enamides stereoselectively (Scheme 22).

**Scheme 23:**
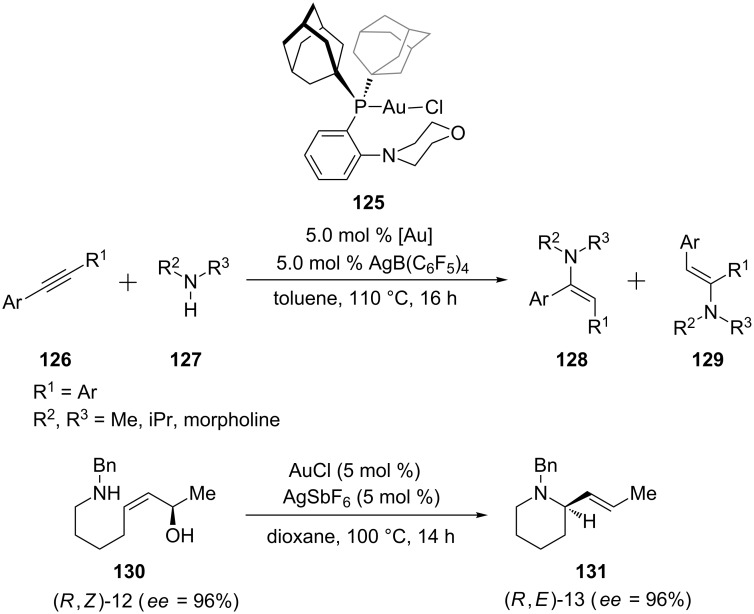
Gold-catalyzed inter- and intramolecular amination of alkynes and alkenes.

Hesp and co-workers have identified a gold pre-catalyst **125** featuring a P,N-ligand that has significantly extended the substrate scope and synthetic utility of alkyne hydroamination [60]. The hydroamination of unsymmetrical internal aryl acetylenes **126** with dialkylamines **127** has been achieved with synthetically useful regioselectivities. In addition to intermolecular addition, Mukherjee and Widenhoefer recently reported a gold(I)-catalyzed intramolecular amination of allylic alcohols **130** with alkylamines (Scheme 23) [61].

#### 3.2 Imines as nucleophiles

**Scheme 24:**
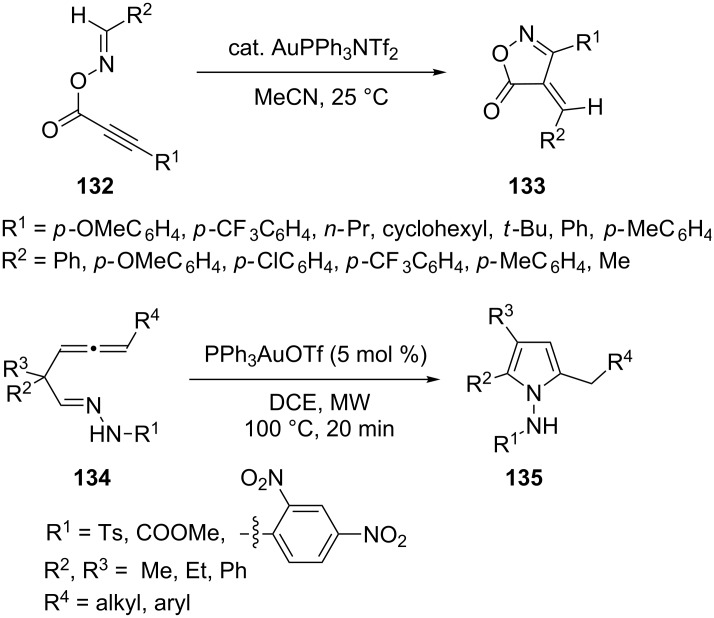
Gold-catalyzed cycloisomerization of *O*-propioloyl oximes and β-allenylhydrazones.

Gold-catalyzed cyclizations of *O*-propioloyl oximes via C–N bond formation followed by arylidene group transfer were developed as a method for the preparation of 4-arylidene isoxazol-5(4*H*)-ones [62]. For example, (*E*)-benzaldehyde *O*-3-phenylpropioloyl oxime **132** was reacted in acetonitrile at 25 °C in the presence of AuPPh_3_NTf_2_ (5 mol %) to give 4-benzylidene-3-phenylisoxazol-5(*4H*)-one **133** in 90% yield. An efficient synthesis of multi-substituted *N*-aminopyrroles **135** via gold(I)-catalyzed cyclization of β-allenylhydrazones **134** was developed by Benedetti and co-workers (Scheme 24) [63]. This intramolecular cyclization method can be applied to both alkyl- or aryl-substituted allenes and involves mild conditions and short reaction times.

#### 3.3 Amides, sulfamides and ureas as nucleophiles

**Scheme 25:**
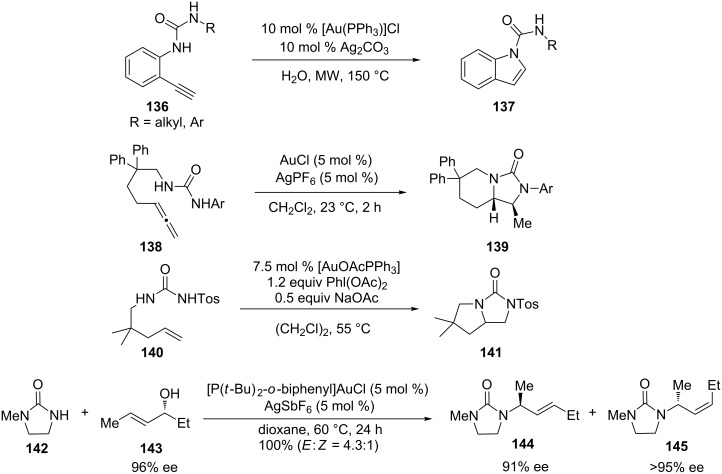
Intra- and intermolecular amination with ureas.

Using AuPPh_3_Cl/Ag_2_CO_3_-catalyzed 5-endo-dig cyclization in water under microwave irradiation, our group developed a fast and green route to prepare indole-1-carboxamides **137** from *N'*-substituted *N*-(2-alkynylphenyl)ureas **136** (Scheme 25) [64]. A variety of functional groups including *N'*-aryl, alkyl, heterocyclic, various *N*-substituted-2-ethynylphenyl and *N*-(2-ethynylpyridin-3-yl)ureas, are tolerated and gives moderate to high yields of the desired products.

In another study [65], bicyclic imidazolidin-2-ones **139** were obtained via gold(I)-catalyzed intramolecular dihydroamination of allenes with *N,N′*-disubstituted ureas **138**. Iglesias et al. reported a complimentary diamination of alkenes **140** with homogeneous gold catalysts [66]. The key step is an intramolecular alkyl–nitrogen bond formation from a gold(III) intermediate. Besides the intramolecular addition of ureas, Widenhoefer’s group reported a gold(I)-catalyzed intermolecular amination of allylic alcohols **143** with cyclic ureas **142** (Scheme 25) [67].

**Scheme 26:**
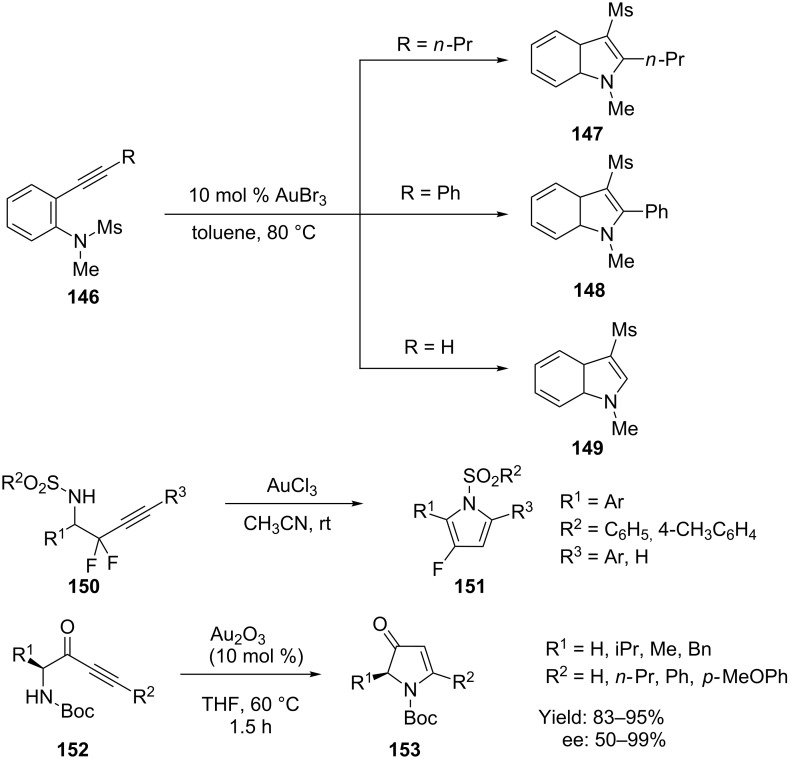
Gold-catalyzed cyclization of *ortho*-alkynyl-*N*-sulfonylanilines and but-3-yn-1-amines.

Gold-catalyzed reactions of *ortho*-alkynyl-*N*-sulfonylanilines **146** produced the corresponding 3-sulfonylindoles in good to high yields (Scheme 26). Nakamura and co-workers synthesized 3-mesyl-1-methyl-2-propylindole **147**, 3-mesyl-1-methyl-2-phenylindole **148**, and 3-mesyl-1-methylindole **149** from *N*-mesyl-*N*-methyl-2-(1-pentynyl)aniline, *N*-mesyl-*N*-methyl-2-(phenylethynyl)aniline, and 2-ethynyl-*N*-mesyl-*N*-ethylaniline in moderate to high yield with AuBr_3_ as the catalyst [68]. Surmont and co-workers later explored a similar strategy for the synthesis of 2-aryl-3-fluoropyrroles **151** [69]. Gouault et al. reported a gold-catalyzed approach to synthesize substituted pyrrolin-4-ones **153** from 1-aminobut-3-yn-2-one analogs **152** under mild conditions [70]. The use of gold(III) oxide as catalyst allows moderate to total stereo control during the cyclization.

**Scheme 27:**
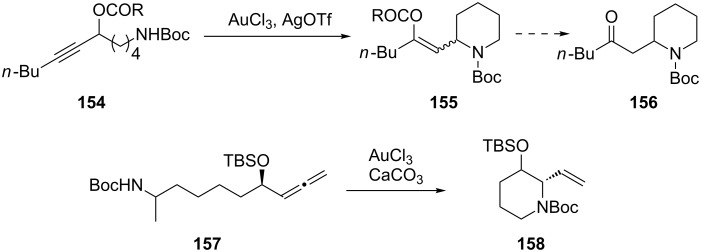
Gold-catalyzed piperidine ring synthesis.

Huang et al. has developed an efficient gold-catalyzed method to access piperidinyl enol esters **155** and piperidinyl ketones **156** under mild reaction conditions from ε-*N*-protected propargylic esters **154** [71]. This intramolecular piperidine cyclization methodology shows different reactivity and substrate applicability compared with the former intermolecular nucleophilic addition. The mechanism speculated by the authors involves a gold-catalyzed intramolecular rearrangement followed by nucleophilic attack of the Boc-protected nitrogen atom. A similar method to synthesize the 2-vinylpiperidin-3-ol **158** by a highly stereoselective gold-catalyzed allene cyclization has been reported (Scheme 27) [72].

**Scheme 28:**
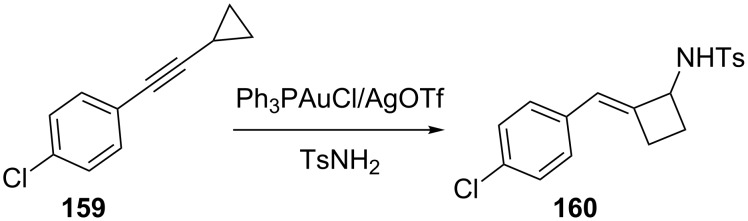
Ring expansion of alkylnyl cyclopropanes.

The ring expansion of cyclopropane derivatives provides a powerful method to construct synthetically useful four-membered carbocycles. Ye et al. reported a new type of gold(I)-catalyzed ring expansion of an non-activated alkynylcyclopropane/sulfonamide to obtain (*E*)-2-alkylidenecyclobutanamines [73]. For example, treatment of alkynylcyclopropane **159** with TsNH_2_ and 5 mol % PPh_3_AuCl/5 mol % AgOTf in dichloroethane at 80 °C gave alkylidenecyclobutanamine **160** in 65% yield as a single olefin isomer (Scheme 28).

**Scheme 29:**
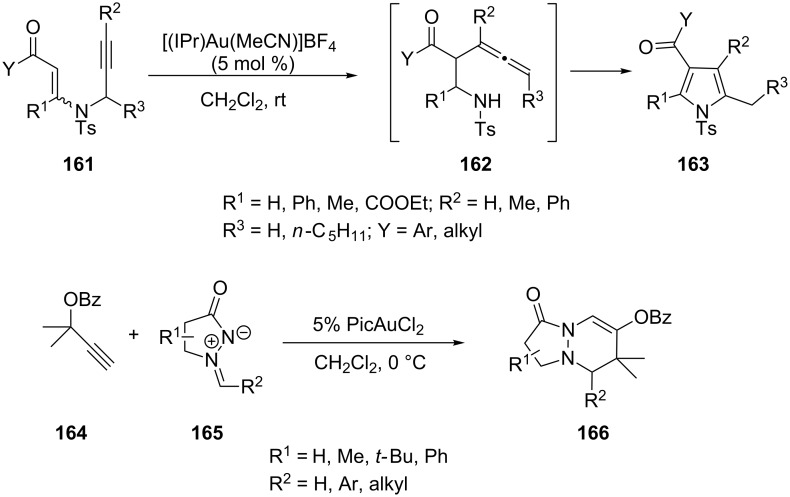
Gold-catalyzed annulations of *N*-propargyl-β-enaminones and azomethine imines.

The formation of tri- and tetrasubstituted pyrroles **163** [74] via cationic *N*-heterocyclic carbene–gold(I) complex catalyzed amino Claisen rearrangement of *N*-propargyl-β-enaminone derivatives **161** and the cyclization of α-allenyl-β-enaminone intermediates has been developed by Saito and co-workers (Scheme 29) [75]. Toste’s group has reported a novel gold(III)-catalyzed [3 + 3]-annulation of azomethine imines **165** with propargyl esters **164**. Substitution of the β-position of the pyrazolidinone generally provides the bicyclic product **166** with high *cis* selectivity, which is determined during ring closing rather than in the formation of allyl–gold intermediate [76].

**Scheme 30:**
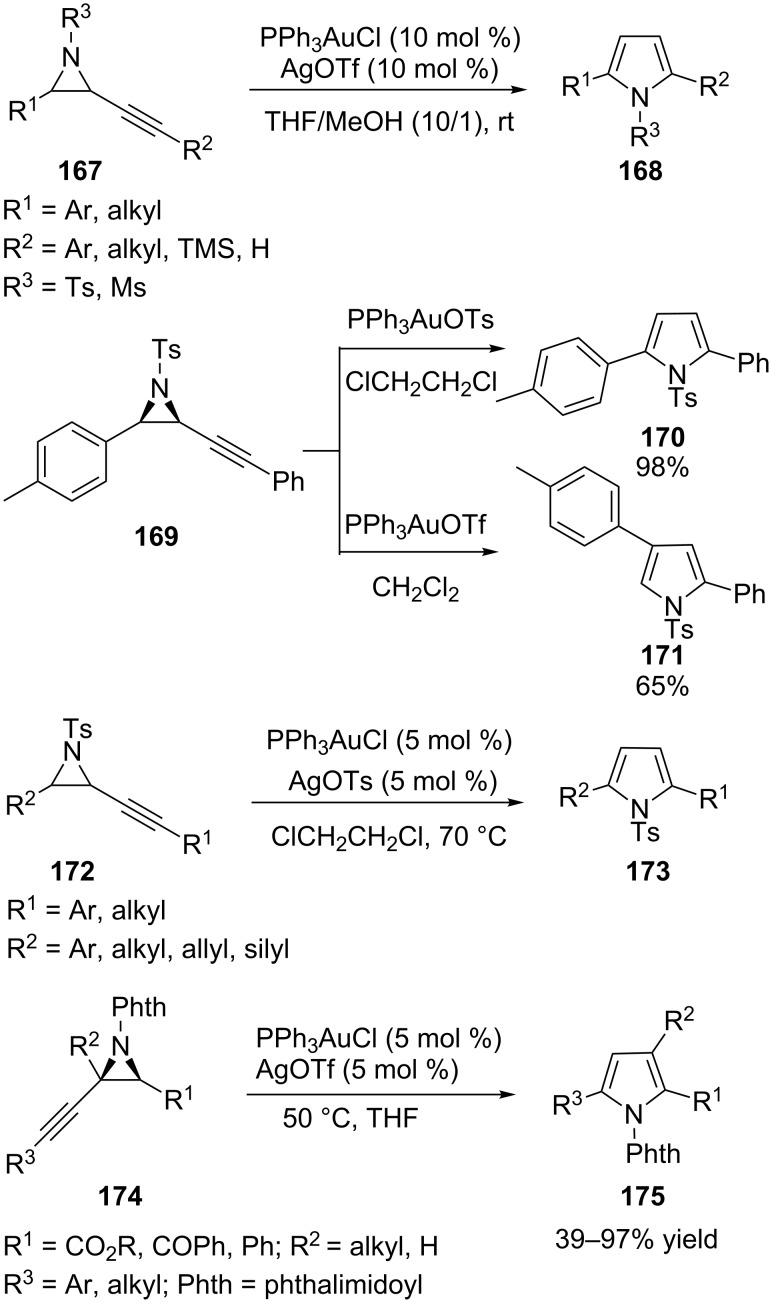
Gold(I)-catalyzed cycloisomerization of aziridines.

Gold-catalyzed cycloisomerization reaction of alkynyl aziridines **167** can give 2,5-disubstituted pyrroles **168** in high yields [77]. However, in some cases, aryl-substituted *N*-tosyl alkynyl aziridines **169** undergo a gold-catalyzed ring expansion to afford 2,5-substituted or 2,4-substituted pyrrole products [78]. Interestingly, the reaction pathway is determined by the counter ion of the gold catalyst. The formation of 2,5-substituted pyrroles **170** proceeds with PPh_3_AuOTs as the catalyst whilst a novel reaction pathway is accessed on changing the catalyst system to PPh_3_AuOTf and leads to 2,4-substituted pyrroles **171**. Recently, the same group reported an efficient and selective synthesis of 2,5-substituted pyrroles **173** by gold-catalyzed ring expansion of alkynyl aziridines **172** [79]. In this study a combination of Ph_3_PAuCl and AgOTs generates a catalyst system that provides clean cycloisomerisation reactions. Similarly, *N*-Phth pyrrroles **175** are obtained via gold-catalyzed cycloisomerization of *N*-Phth alkynyl aziridines **174** (Scheme 30) [80].

**Scheme 31:**
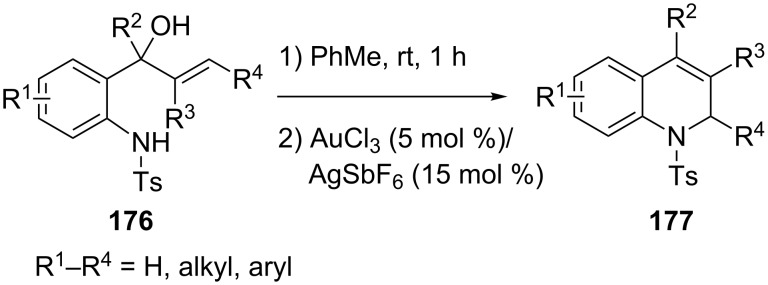
AuCl_3_/AgSbF_6_-catalyzed intramolecular amination of 2-(tosylamino)phenylprop-1-en-3-ols.

Chan’s group developed an efficient synthetic route to 1,2-dihydroquinolines **177** via AuCl_3_/AgSbF_6_-catalyzed intramolecular allylic amination of 2-(tosylamino)phenylprop-1-en-3-ols **176** (Scheme 31) [81]. The mechanism is suggested to involve activation of the alcohol substrate by the AuCl_3_/AgSbF_6_ catalyst and ionization of the starting material, which causes intramolecular nucleophilic addition of the sulfonamide unit to the allylic cation moiety and construction of a 1,2-dihydroquinoline.

**Scheme 32:**
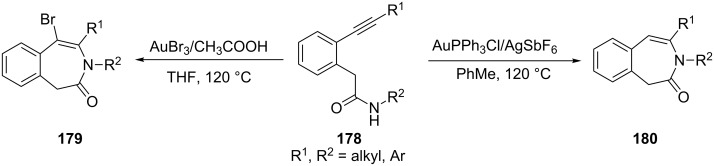
Gold-catalyzed cyclization via a 7-endo-dig pathway.

Our group also discovered that a regioselective hydroamidation of 2-(1-alkynyl)phenylacetamides **178** could be achieved with AuPPh_3_Cl/AgSbF_6_ as the catalyst and gave 3-benzazepin-2-ones **180** via 7-endo-dig pathway [82]. Moreover, a AuBr_3_-mediated transformation of 2-(1-alkynyl)phenylacetamides **178** to 5-bromo-3-benzazepin-2-ones **179** was discovered, which indicated that the gold catalyst not only played an activation role but also acted as a reactant in the reaction (Scheme 32).

**Scheme 33:**
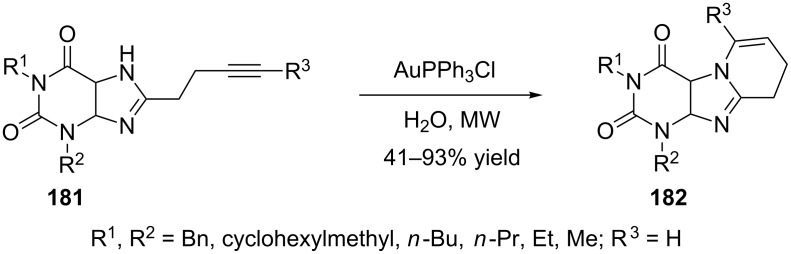
Gold-catalyzed synthesis of fused xanthines.

A simple, convenient, and green synthetic approach to diverse fused xanthines **182** has also been developed by gold-complex catalyzed intramolecular hydroamination of terminal alkynes **181** under microwave irradiation in aqueous media (Scheme 33). This transformation is atom-economical and has high functional group tolerance [83].

#### 3.4 Nitriles and nitrines as nucleophiles

**Scheme 34:**
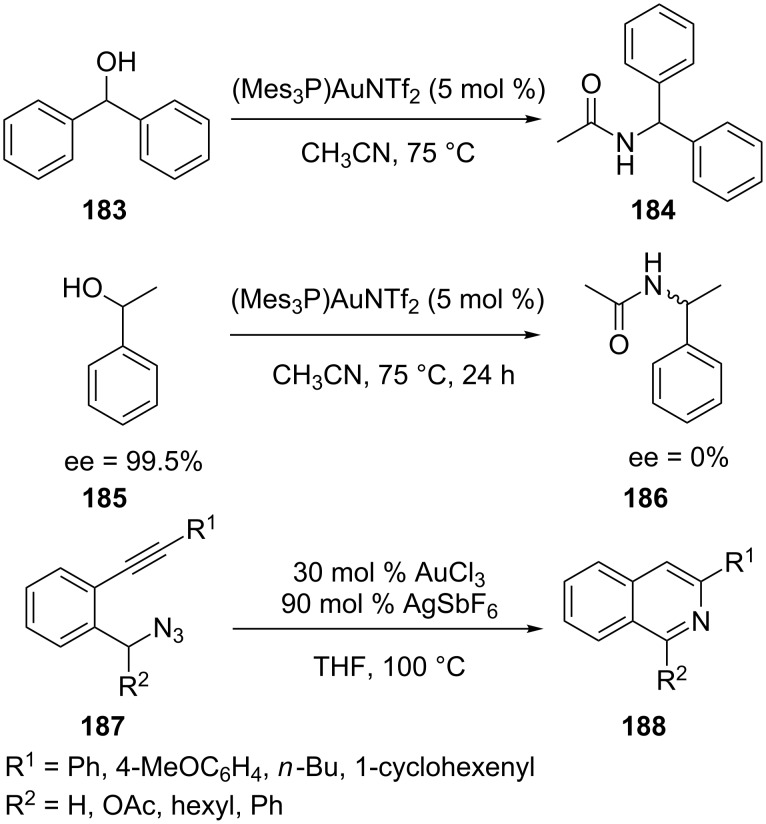
Gold-catalyzed synthesis of amides and isoquinolines.

Ibrahim et al. reported a new and mild method for the synthesis of amide **184** from readily available benzhydrol **183** and nitriles catalyzed by a gold(I)-complex with a trimesitylene ligand [84]. Mechanistic control experiments with chiral alcohol **185** prove the intermediacy of carbenium ions. Further studies with not readily ionizable alcohols also indicate that for the benzhydrols the carbenium ions and gold(I)-hydroxy complexes are intermediates (Scheme 34). Yamamoto’s group reported that intramolecular cyclization of 2-alkynylbenzyl azides **187** in the presence of AuCl_3_ and AgSbF_6_ in THF under pressure at 100 °C gives the corresponding isoquinolines **188** in good yields [85].

### 4 Gold-catalyzed C–C bond formations

The formation of carbon–carbon bonds by using various transition metals such as Pd, Ni, Ru, Rh has been extensively investigated and is well documented in the literature. Recent years have witnessed a tremendous growth in the number of gold-catalyzed highly selective chemical transformations. Although gold was considered to be an inert metal for a long time, its ability to behave as a soft Lewis acid has only been recently recognized. Such a property allows it to activate unsaturated functionalities such as alkynes, alkenes, and allenes, to create C–C bonds under extremely mild conditions [15].

#### 4.1 Intermolecular coupling

**Scheme 35:**
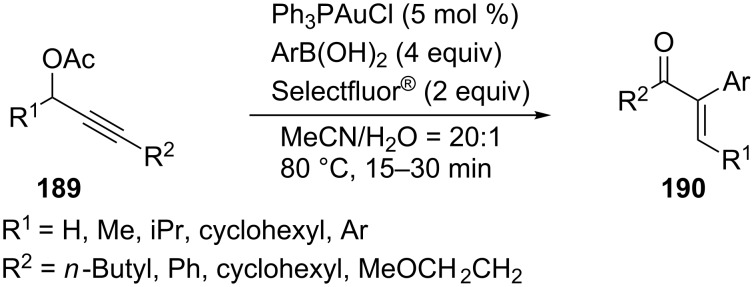
Gold-catalyzed oxidative cross-coupling reactions of propargylic acetates.

An unprecedented homogeneous gold-catalyzed oxidative cross-coupling which leads to α-arylenones **190** from propargylic acetates **189** and arylboronic acids has been developed by Zhang’s group (Scheme 35) [86]. This cross-coupling reaction reveals the synthetic potential of Au(I)/Au(III) catalytic cycles.

**Scheme 36:**
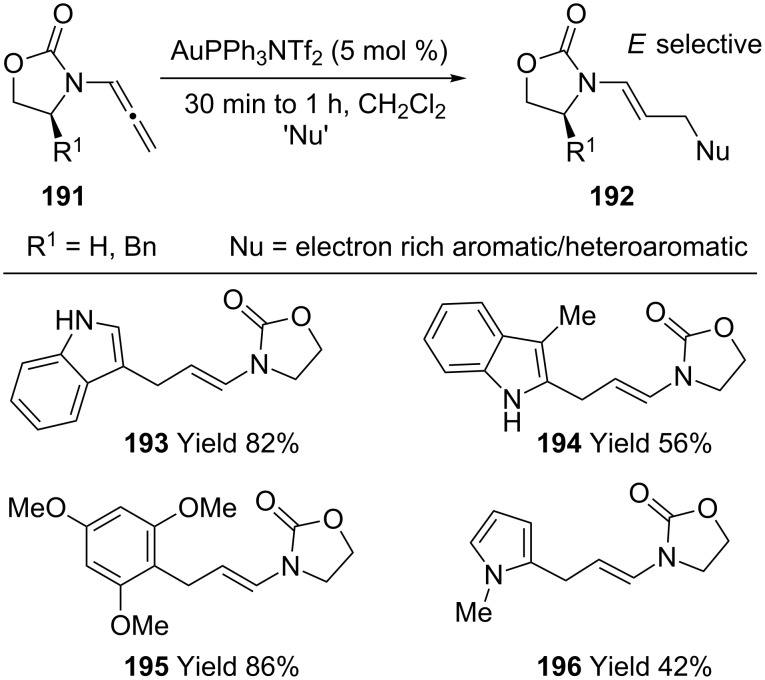
Gold-catalyzed nucleophilic addition to allenamides.

Kimber reported a facile and mild synthesis of enamides (**193**–**196**) by a gold-catalyzed nucleophilic addition to allenamides **191** (Scheme 36) [87]. For example, treatment of allenamide and 1-methylindole with 5.0 mol % of PPh_3_AuNTf_2_ in CH_2_Cl_2_ at room temperature gave the corresponding enamide in 83% yield.

**Scheme 37:**
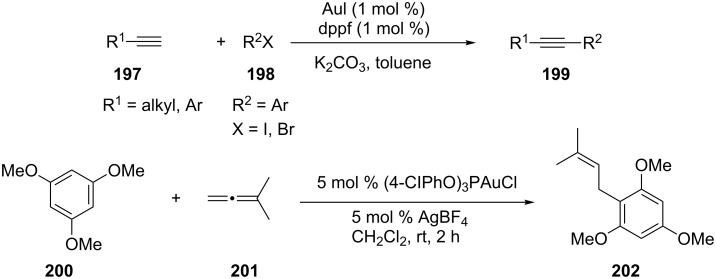
Gold-catalyzed direct carbon–carbon bond coupling reactions.

Gold-catalyzed direct carbon–carbon bond coupling reactions have been less explored [88–89]. In 2008, Li et al. reported a gold(I) iodide catalyzed Sonogashira reaction [88]. Terminal alkynes **197** reacted smoothly with aryl iodides and bromides **198** in the presence of 1 mol % AuI and 1 mol % dppf to generate the corresponding cross-coupling products **199** in good to excellent yields (Scheme 37). Another direct carbon–carbon bond coupling reaction was reported by Tarselli and co-workers [90]. In their study, the addition of nucleophilic methoxyarenes **200** to allenes **201** proceeded at room temperature in dichloromethane with a catalytic amount of phosphite–gold(I) pre-catalyst and a silver additive. Notably, the addition is regioselective for the allene terminus, and generates (*E*)-allylation products **202**.

**Scheme 38:**
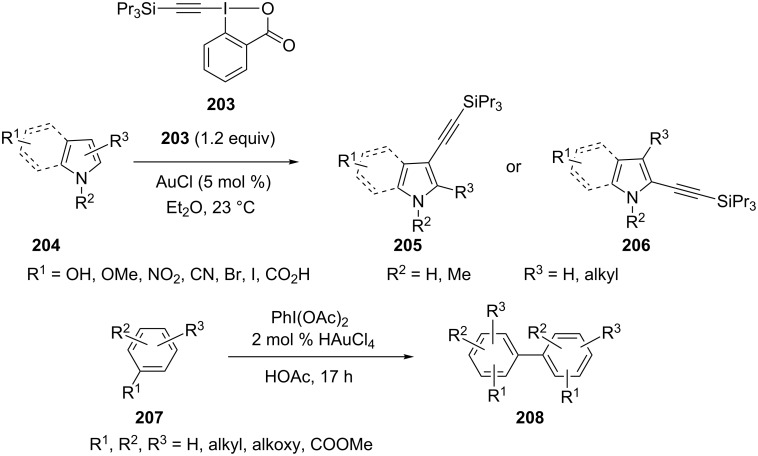
Gold-catalyzed C−H functionalization of indole/pyrrole heterocycles and non-activated arenes.

The direct C–H functionalization of indoles or pyrroles is an efficient method for the introduction of vinyl and aryl groups. A gold-catalyzed direct alkynylation of indole and pyrrole heterocycles **204** with a benziodoxolone-based hypervalent iodine reagent **203** has been developed [91]. The functional group tolerance was greatly increased when compared with direct alkynylation of indoles reported previously. Kar et al. reported a general gold-catalyzed direct oxidative homo-coupling of non-activated arenes **207** (Scheme 38). The reaction protocol tolerates a wide range of functional groups [92]. All halogens survive the reaction, which provides the potential for further reactions.

#### 4.2 Rearrangements and ring enlargement

**Scheme 39:**
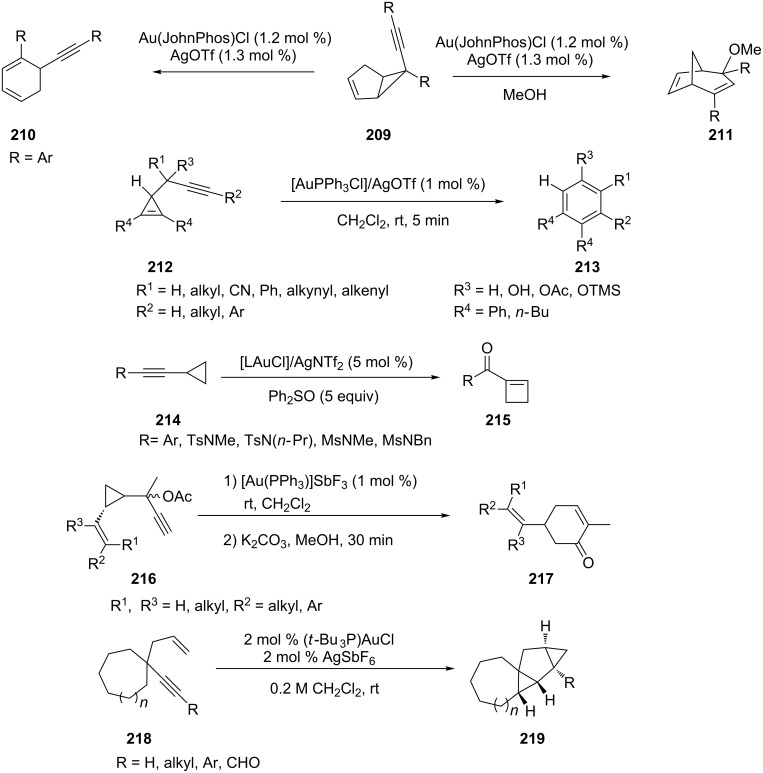
Gold-catalyzed cycloisomerization of cyclic compounds.

A gold-catalyzed rearrangement of 6-alkynylbicyclo[3.1.0]hexen-2-enes **209** has been developed [93]. In this reaction, divergent structural rearrangements are observed in the absence/presence of nucleophiles. The process results in a novel five-to-six-membered ring expansion that involves cleavage of the bridging C–C bond and a formal [1,2]-alkynyl shift. Li et al. reported the first gold-catalyzed reaction of propargylcyclopropene systems **212** which affords benzene derivatives **213** in high yields [94] (Scheme 39).

Only few efficient methods have emerged for the synthesis of cyclobutane derivatives, which are important structural units in several natural products. Li et al. reported a novel gold-catalyzed oxidative ring-expansion of non-activated cyclopropylalkynes using Ph_2_SO as an oxidant [95]. Various alkynylcyclopropane derivatives **214** have been converted to cyclobutenyl ketones **215** in moderate to high yields under optimal conditions. Zou et al. has developed a versatile approach to 5-, 6-, and 7-membered carbocycles via the gold-catalyzed cycloisomerization of cyclopropyl alkynyl acetates [96]. The homo-Rautenstrauch rearrangement of 1-cyclopropylpropargylic esters **216** gave cyclohexenones **217** under mild conditions. Toste’s group reported a gold(I)-catalyzed sequential cycloisomerization/sp^3^ C–H bond functionalization (Scheme 39) of 1,5-enynes **218** and 1,4-enallenes to yield tetracyclododecane **219** and tetracyclotridecane derivatives, respectively [97]. These transformations represent rare examples of sp^3^ C–H bond insertion via a cationic gold(I)–carbenoid intermediate.

#### 4.3 Cycloadditions

**Scheme 40:**
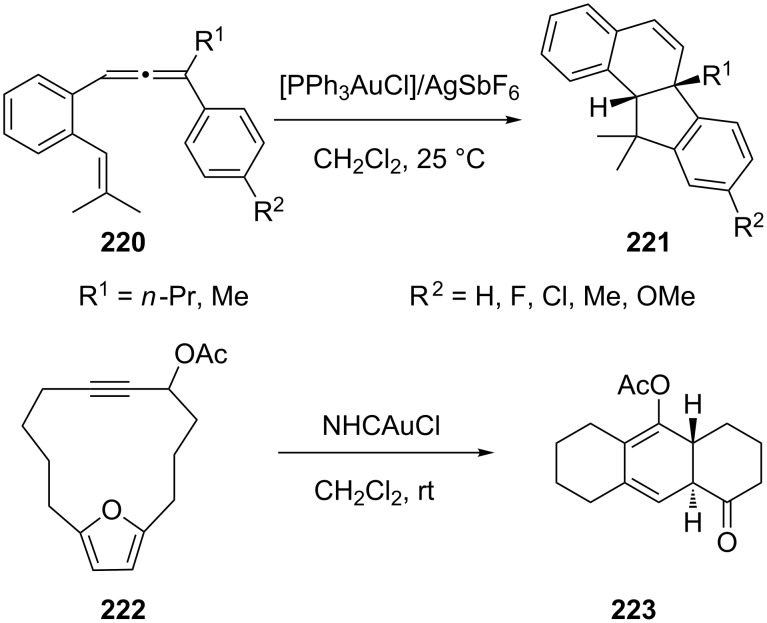
Gold-catalyzed cycloaddition of 1-aryl-1-allen-6-enes and propargyl acetates.

Intramolecular [M + N]-type cycloaddition reactions are powerful tools for accessing complex molecular frameworks [98]. Several gold-catalyzed [3 + 2] [99], [4 + 2] [100–105], and [4 + 3] [106–108] cycloaddition reactions have been developed in last 3 years. Treatment of 1-aryl-1-allen-6-enes **220** with [PPh_3_AuCl]/AgSbF_6_ (5 mol %) in CH_2_Cl_2_ at 25 °C led to intramolecular [3 + 2] cycloadditions to afford *cis*-fused dihydrobenzo[*a*]fluorenes **221** efficiently and selectively [99]. As pointed out by the researchers, the reactions proceeded with the initial formation of *trans*/*cis* mixtures of 2-alkyl-1-isopropyl-2-phenyl-1,2-dihydronaphthalene cations, which were converted into the desired *cis-*fused cycloadducts through the combined action of a gold catalyst and a Brønsted acid. Gung and co-workers developed a 3,3-rearrangement/transannular [4 + 3] cycloaddition reaction (Scheme 40) in the presence of either a Au(I) or Au(III) catalyst [109]. In these reactions, the regiochemistry of the product **223** is controlled by the position of the acetoxy group in the starting material **222**, while the stereochemistry of the reaction depends on the ring size.

**Scheme 41:**
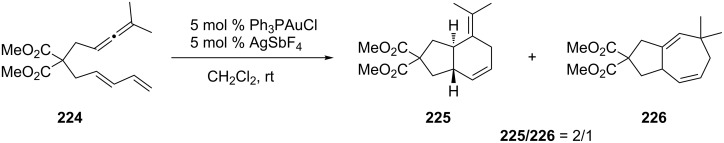
Gold(I)-catalyzed cycloaddition with ligand-controlled regiochemistry.

In some gold(I)-catalyzed cycloaddition reactions, regiochemistry of the product is controlled by the ligand [100–101]. For example, the triphenylphosphinegold(I)-catalyzed reaction of allene–diene **224** provided a 2:1 mixture of the [4 + 3] and [4 + 2] cycloadducts (**225** and **226**) [101]. The selectivity was improved to 96:4 in favor of the [4 + 3] cycloadduct when di-*tert*-butylbiphenylphosphinegold(I) was employed as the catalyst. On the other hand, the use of arylphosphitegold(I) complexes exclusively produced the formal [4 + 2] cycloaddition product in very good yield (Scheme 41).

Enynes [110–116], diynes [117–120], allenynes [121–128], and dienes [129–131] are common substrates for intramolecular cycloaddition reactions.

**Scheme 42:**
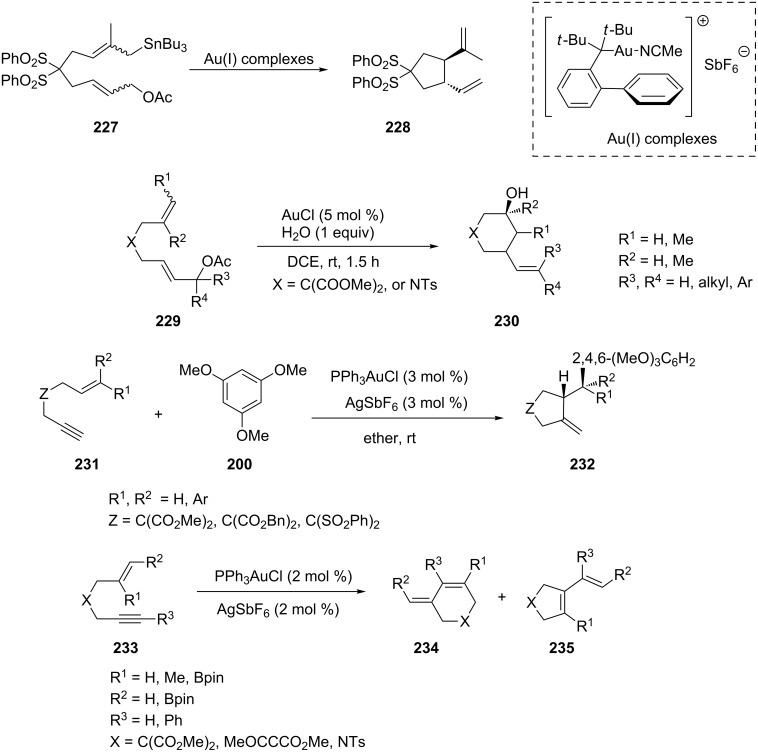
Gold(I)-catalyzed cycloaddition of dienes and enynes.

Porcel et al. found that cationic Au(I) complexes are the most efficient catalysts for the intramolecular coupling of allyl acetates with allylstannanes (compound **227**) [129]. Zhu and co-workers reported a gold-catalyzed carbocyclization of dienyl acetates **229** to construct multi-functionalized 3-vinylcyclohexanol derivatives **230** [130]. The reaction proceeded through the nucleophilic addition of the alkene to the allylic cation via a 6-endo-trig process. The structure of the substrate affected the configurational orientation of the allylic cation in a boat-like transition state, which led to either *trans-*cyclohexanols or *cis-*piperidine derivatives. Some functionalized carbo- and heterocycles **232** were synthesized via gold-catalyzed cycloisomerization reactions of enynes **231** [110]. The PPh_3_AuCl/AgSbF_6_ catalytic system promotes a Friedel–Crafts type addition of electron-rich aromatic and heteroaromatic derivatives to the non-activated alkene followed by a C–C bond cyclization reaction. The carbon, oxygen and nitrogen tethered 1,6-enynes react smoothly with methoxy substituted benzenes, indoles, pyrroles and furans as nucleophilic partners (Scheme 42). The cycloisomerization reactions of boronated enynes **233** was achieved with gold(I) complexes generated from a mixture of gold and silver salts [111]. Both, alkynyl and alkenyl pinacol boronates were tolerated. The ratio of the different *endo*- and *exo-*products was heavily dependent on the position of the boronate functionality (Scheme 42).

**Scheme 43:**
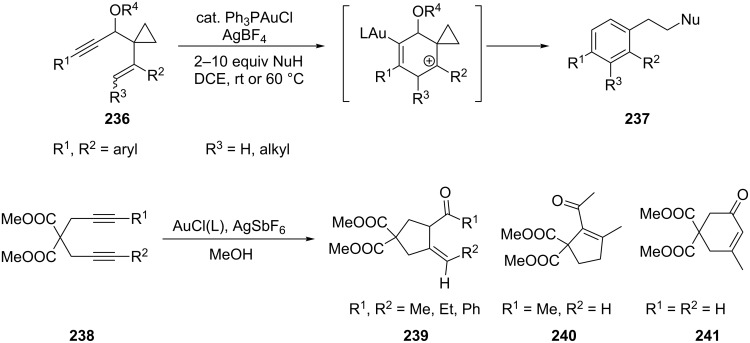
Gold-catalyzed intramolecular cycloaddition of 3-alkoxy-1,5-enynes and 2,2-dipropargylmalonates.

Li et al. reported a gold-catalyzed benzannulation of 3-alkoxy-1,5-enynes **236** to produce functionalized benzenes **237** [112]. The reaction occurs selectively through a 6-endo-dig pathway to give tri- and tetrasubstituted benzenes efficiently. Cyclization reactions of 1,6-diynes (2,2-dipropargylmalonates **238**) could be achieved with gold(I) catalysts. Disubstituted 1,6-diynes furnished the (*Z*)-cyclopentylidene derivative **239** stereoselectively [117]. Monosubstituted terminal diyne afforded the cyclopentene derivative **240**, while the diterminal 1,6-diyne produced a cyclohexenone derivative **241** (Scheme 43).

**Scheme 44:**
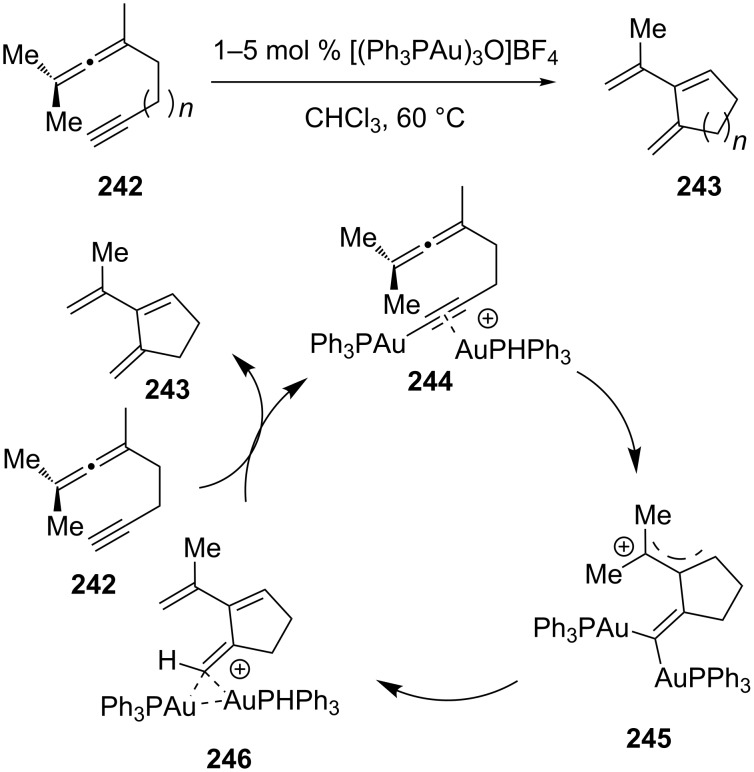
Gold-catalyzed intramolecular cycloaddition of 1,5-allenynes.

Cheong and co-workers demonstrated that 1,5-allenynes **242** could be transformed to cross-conjugated trienes **243** via rearrangement with [(Ph_3_PAu)_3_O]BF_4_ as the catalyst [121]. Computational results indicated that the ene-reaction proceeded through a unique nucleophilic addition of an allene double bond to a cationic phosphine-gold(I)-complexed phosphine-gold(I) acetylide, followed by a 1,5-hydrogen shift (Scheme 44).

**Scheme 45:**
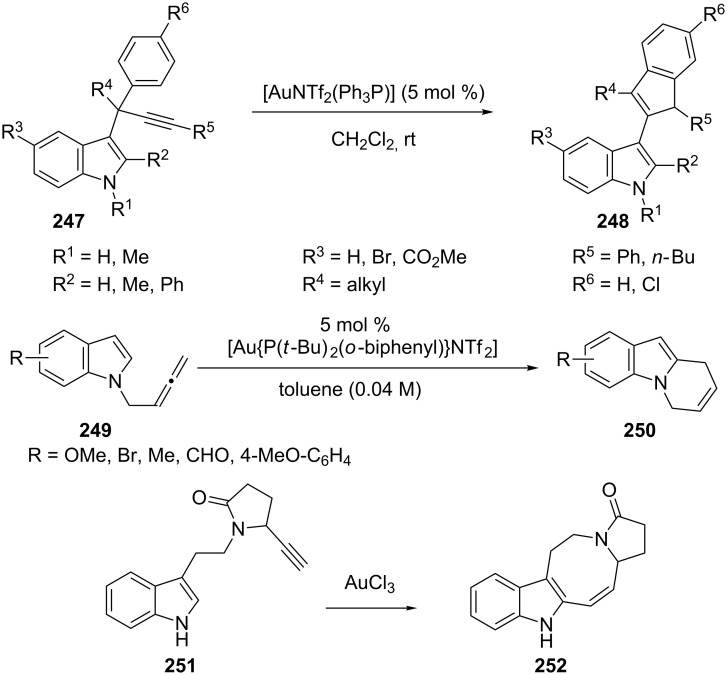
Gold(I)-catalyzed cycloaddition of indoles.

A range of indole based cycloaddition products were obtained by concerting the initial regioselective site-selective indole attack (C3 position) to the C–C multiple bonds [132–134]. In the case of gold(I)-catalyzed reactions initiated by 1,2-indole migrations [132], the starting material, indole **247**, was converted to an intermediate with [AuNTf_2_(Ph_3_P)]. Intramolecular attack of the indole on the activated alkyne gives the vinyl–gold complex, which is transformed into the gold carbene complex through a 1,2-migration of the indole. Further intramolecular nucleophilic attack of the phenyl group on the carbene carbon center, followed by a re-aromatization step and subsequent protodemetalation, affords **248** as the final product. Treatment of *N*-tethered 2,3-butadienyl-1*H*-indole **249** with di-*tert*-butyl(*o*-biphenyl)phosphine and AuNTf_2_ led to 6-*endo* cyclization [133]. The methodology was applied in a direct synthesis of the relevant 6,9-dihydropyrido[1,2-*a*]-1*H*-indole core **250**. A similar strategy was adopted by Ferrer and co-workers [134], who prepared the 1*H*-azocino[5,4-*b*]indole skeleton **252** of the lundurines by the 8-endo-dig cyclization of the alkynylindole **251** using gold(III) chloride as the catalyst (Scheme 45).

**Scheme 46:**
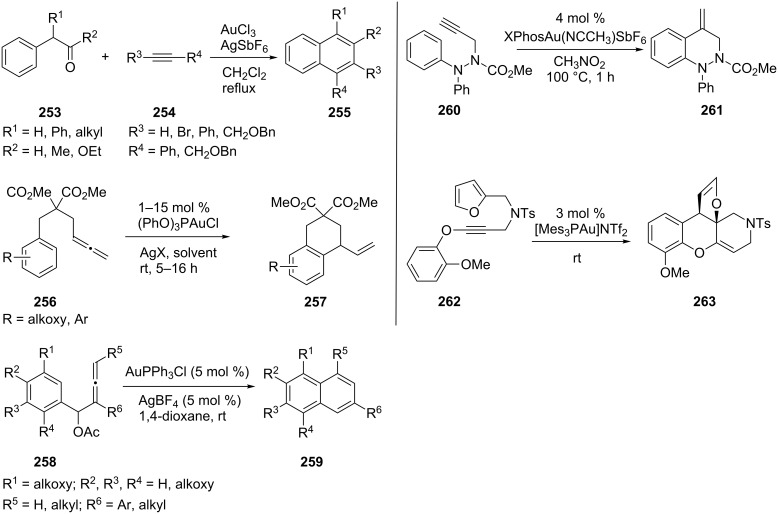
Gold-catalyzed annulation reactions.

Electron-rich arenes are, in some cases, good nucleophiles [135–136]. An interesting gold-catalyzed electrophilic addition to an arylalkyne for the synthesis of substituted naphthalenes **255** has been developed [137]. Tarselli et al. reported a gold(I)-catalyzed intramolecular hydroarylation of allenes [138]. Gold(I) complexes react with 4-allenylarenes **256** in an *exo* fashion to furnish vinyl-substituted benzocycles **257**. Interestingly, if 1-arylbuta-2,3-dienyl acetate **258** was used as the substrate, naphthalenes **259** are formed through a AuPPh_3_Cl catalyzed cyclization reaction [139]. Using gold complex [XPhosAu(NCCH_3_)SbF_6_] as the catalyst, Jurberg and Gagosz prepared the cinnoline derivatives **261** by the hydroarylation of *N*-propargyl-*N*-arylhydrazines **260** [140]. With the gold complex [Mes_3_PAu]NTf_2_, an alkynyl ether moiety triggered a new reaction mode of furan–yne cyclization and delivered a new class of tetracyclic system **263** rather than a phenol (Scheme 46) [141].

**Scheme 47:**
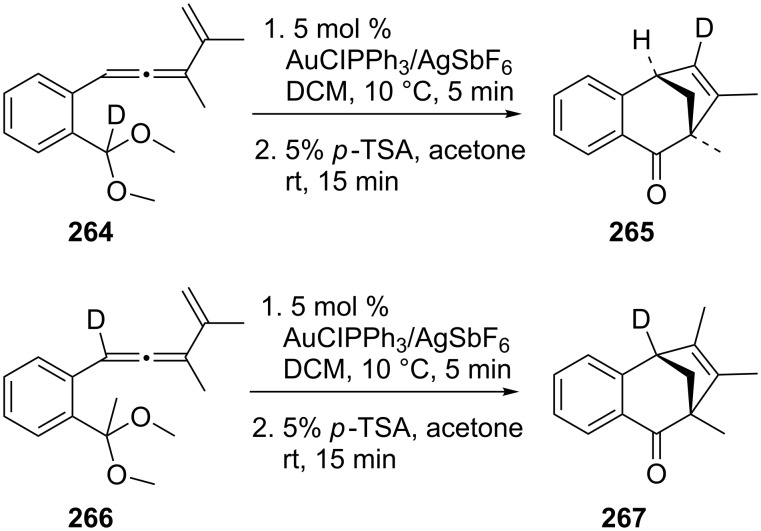
Gold–carbenoid induced cleavage of a sp^3^-hybridized C−H bond.

Insertion of a C–H bond into a metal–carbenoid is a highly useful method for forming a new carbon–carbon bond. An atypical gold–carbenoid induced cleavage of a sp^3^-hybridized C–H bond can be achieved by undergoing 1,3-addition to a vinyl–carbenoid intermediate [142]. The bicyclo[3.2.1]oct-6-en-2-ones **265** and **267** could be synthesized stereoselectively by this method. Deuterium labeling experiments indicated the cyclization involved an unprecedented 1,3-addition of a sp^3^-hybridized C–H bond to the vinyl–carbenoid moiety (Scheme 47).

### 5 Gold-catalyzed tandem reactions

Tandem catalysis refers to the synthetic strategies of modular combination of catalytic reactions into one synthetic operation with minimum workup or change in conditions [143]. The gold-catalyzed tandem reactions have allowed chemists to assemble diverse complex molecular frameworks more conveniently.

#### 5.1 Sequential inter-and intramolecular reactions

**Scheme 48:**
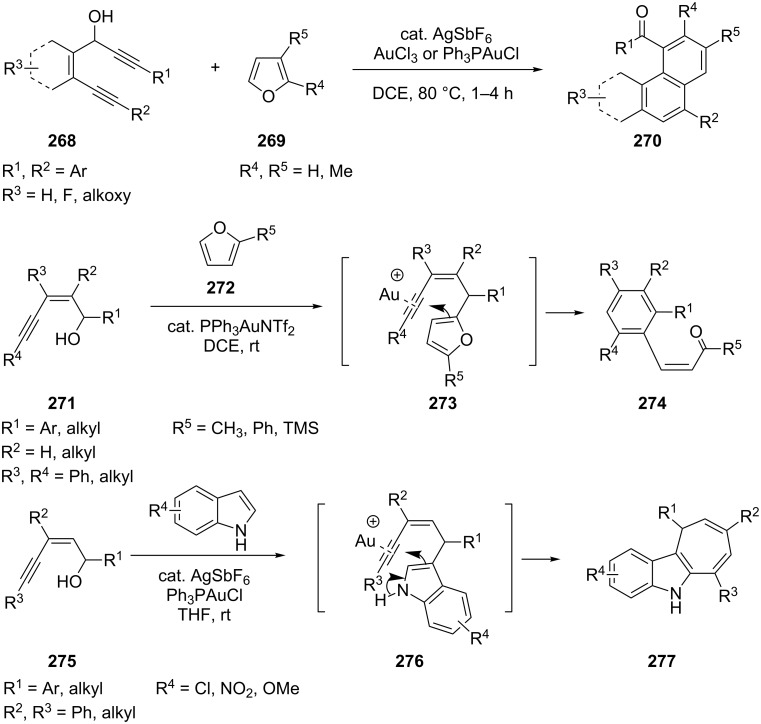
Furan- and indole-based cascade reactions.

Phenanthrenyl ketones are very important subunits in material science and also occur in numerous natural products. A gold-catalyzed cascade Friedel–Crafts/furan–yne cyclization/heteroenyne metathesis was developed for the highly efficient construction of phenanthrene derivatives **270** [144]. Both AuCl_3_ and PPh_3_AuCl are effective catalysts for all the processes in the reaction and a variety of diyne substrates **271** could be used (Scheme 48). Similar strategies [145–146] were applied to synthesize arylated (*Z*)-enones, -enals or dihydrocyclohepta[*b*]indole skeletons **277** by gold-catalyzed cascade Friedel–Crafts/furan (or indole)–alkyne cycloisomerizations (Scheme 48).

**Scheme 49:**
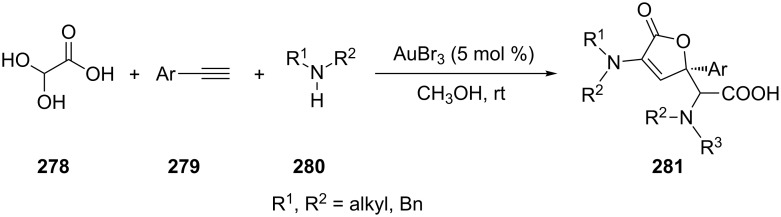
Tandem process using aromatic alkynes.

The polysubstituted butenolides **281** could be obtained through a gold-catalyzed multi-component tandem reaction that involved novel direct alkyne **279**–amine **280**–glyoxylic acid (**278**) coupling, intramolecular cyclization of α-*N*-substituted β-alkynoic acid, and subsequent reaction (Scheme 49) [147].

**Scheme 50:**
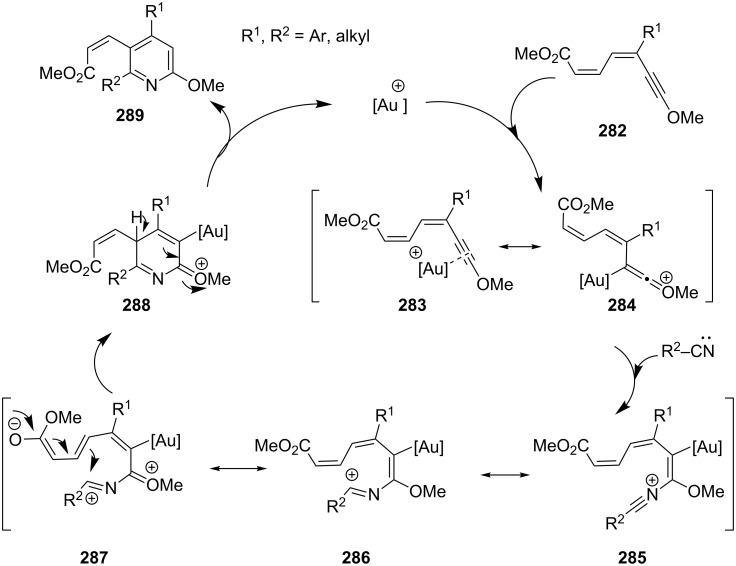
Gold-catalyzed cycloaddition of 1,3-dien-5-ynes.

An intermolecular hetero-dehydro-Diels–Alder reaction between captodative 1,3-dien-5-ynes **282** and non-activated nitriles was introduced by Barluenga and co-workers [148]. The sequence is promoted by both, gold(I) and gold(III) catalysts and leads to the regioselective formation of tetrasubstituted pyridines **289**. The initial coordination of the triple bond to the gold catalyst forms intermediate **284**, followed by the regioselective nucleophilic attack of the nitrile, leading to the formation of **285**. Cyclization may occur through resonance structure **286** or **287** followed by final metal de-coordination (Scheme 50).

#### 5.2 Sequential intramolecular reactions

**Scheme 51:**
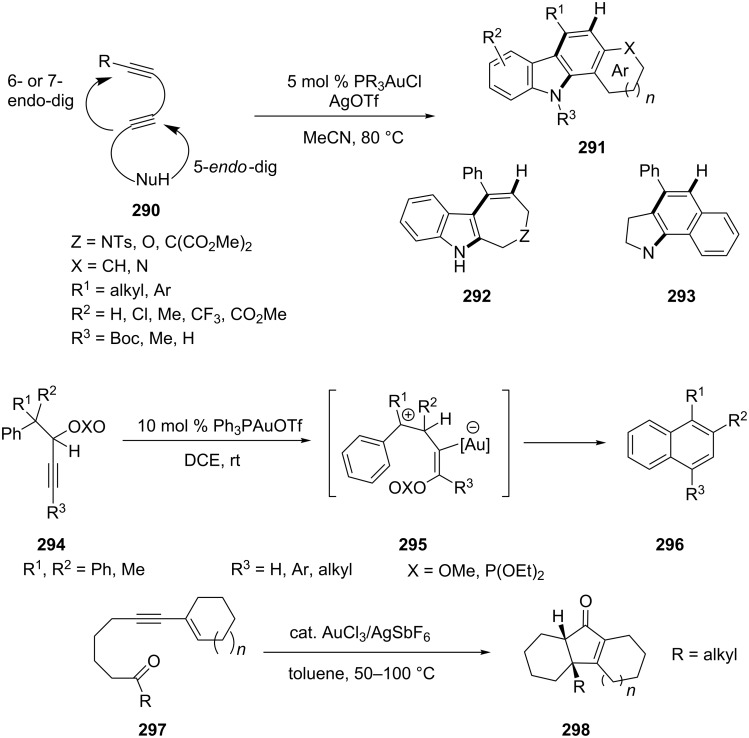
Gold-catalyzed cascade cyclization of diynes, propargylic esters, and 1,3-enynyl ketones.

Sequential intramolecular reactions result in the formation of multi-ring products from a single substrate [149]. In 2010, a concise synthetic method for the generation of fused indoles (**291**–**293**), by a gold-catalyzed cascade cyclization of diynes **290** was developed by Hirano and co-workers [150]. The reaction gave aryl annulated[*a*]carbazoles, dihydrobenzo[*g*]indoles, and azepino- or oxepinoindole derivatives through an intramolecular cascade 5-endo-dig hydroamination followed by a 6- or 7-endo-dig cycloisomerization. Dudnik et al. reported a gold(I)-catalyzed cycloisomerization of propargylic esters **294** which led to unsymmetrically substituted naphthalenes **296** [151]. This cascade reaction involves a tandem sequence of 1,3- and 1,2-migration of two different migrating groups. Jin and Yamamoto prepared the fused tri- and tetracyclic enones **298** through an efficient gold(III)-catalyzed tandem reaction, heteroenyne metathesis, and Nazarov cyclization of 1,3-enynyl ketones **297** [152]. The gold(III) catalyst exhibits dual roles for activating both the alkyne and carbonyl moieties (Scheme 51).

**Scheme 52:**
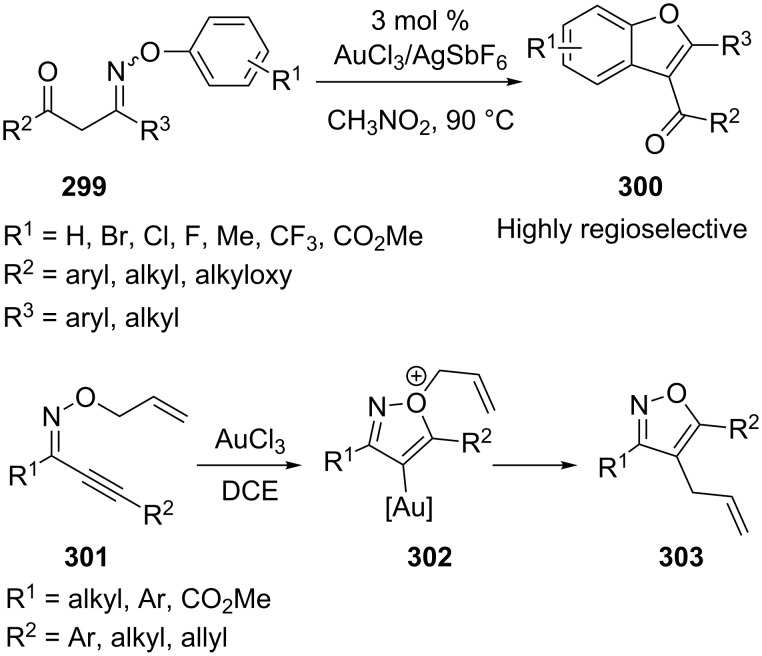
Tandem reaction of β-phenoxyimino ketones and alkynyl oxime ethers.

More recently, Liu et al. developed a gold(III)-catalyzed tandem rearrangement/cyclization reaction of β-phenoxyimino ketone **299 (**produced from *O*-arylhydroxylamines with 1,3-dicarbonyl compounds in situ) to give 3-carbonylated benzofuran derivatives **300** [153]. Trisubstituted isoxazoles **303** were obtained from alkynyl oxime ether **301** through a gold-catalyzed domino reaction involving cyclization and subsequent Claisen-type rearrangement [154]. The presence of additional substituents on the allyl moiety required an increase in catalyst loading and a prolonged reaction time for complete consumption of the substrate (Scheme 52).

**Scheme 53:**
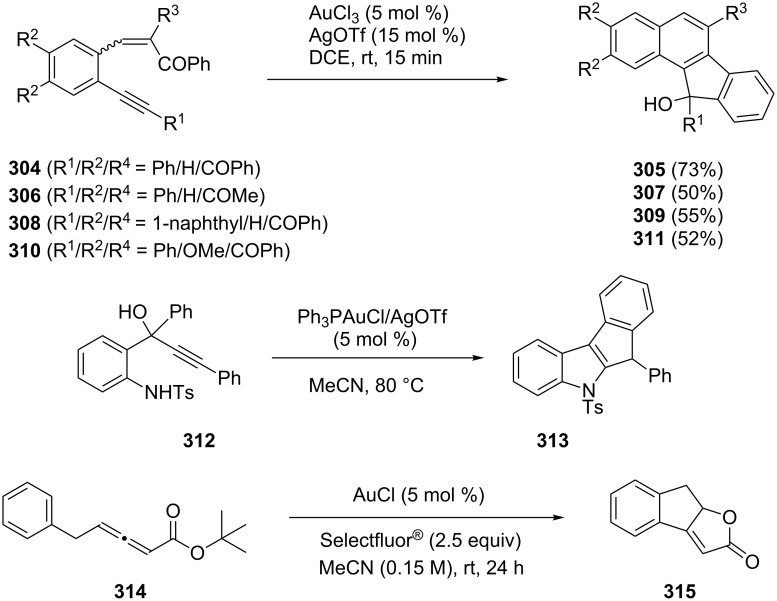
Gold-catalyzed tandem cyclization of enynes, 2-(tosylamino)phenylprop-1-yn-3-ols, and allenoates.

Liu and Zhang have developed a gold-catalyzed region-divergent tandem cationic cyclization/ring expansion terminated by a pinacol rearrangement to produce naphthalen-2(1*H*)-ones or naphthalenes **305**, **307**, **309**, and **311** selectively (Scheme 53) [155]. The synthesis of indole **313** [156] and tricyclic dihydroindenofuranone-type product **315** from 2-(tosylamino)phenylprop-1-yn-3-ol **312** [157] and allenoates **314** [158], respectively, has been reported (Scheme 53). The latter is the first example of a gold-catalyzed intramolecular C–C cross-coupling reaction involving aryl C–H functionalization with Selectfluor^®^ as the oxidant.

**Scheme 54:**
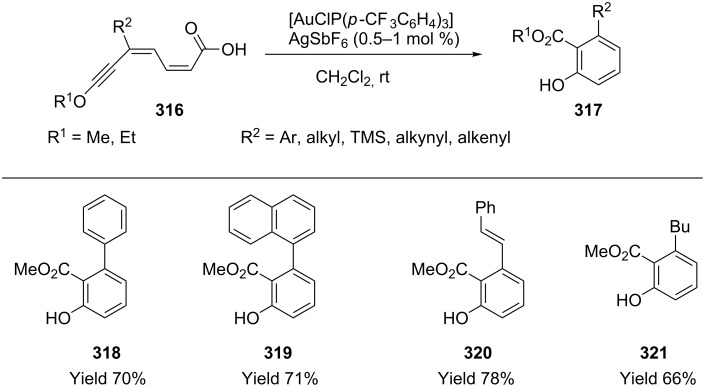
Cyclization of 2,4-dien-6-yne carboxylic acids.

2,4-Dien-6-ynecarboxylic acids **316** undergo gold-catalyzed tandem 1,6-cyclization/decarboxylation to afford 2,3-disubstituted phenols (**318**–**321**) and unsymmetrical bi- and terphenyls (Scheme 54) [159]. The reaction is greatly affected by the electronic properties of dienyne acid. The regioselective 1,6-cyclization/decarboxylation sequence only takes place when a strong electron-donating group is not directly linked to the triple bond.

**Scheme 55:**
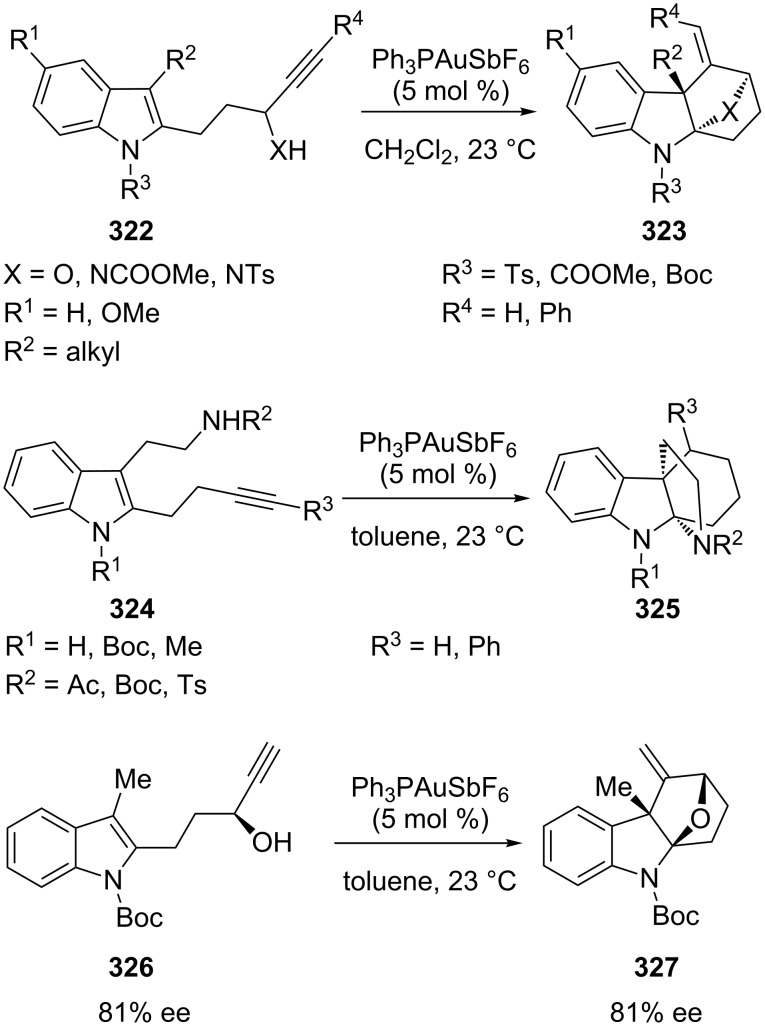
Gold(I)-catalyzed tandem cyclization approach to tetracyclic indolines.

Liu et al. has developed two highly stereoselective cationic gold(I)-catalyzed tandem cyclization reactions of alkynylindoles **322** [160]. The reaction proceeds with remarkable retention of chirality and allows the efficient enantioselective synthesis of polycyclic indolines **327** from the corresponding enantiomerically enriched alkynylindole **326** (Scheme 55).

#### 5.3 Sequential intra-and intermolecular reactions

**Scheme 56:**
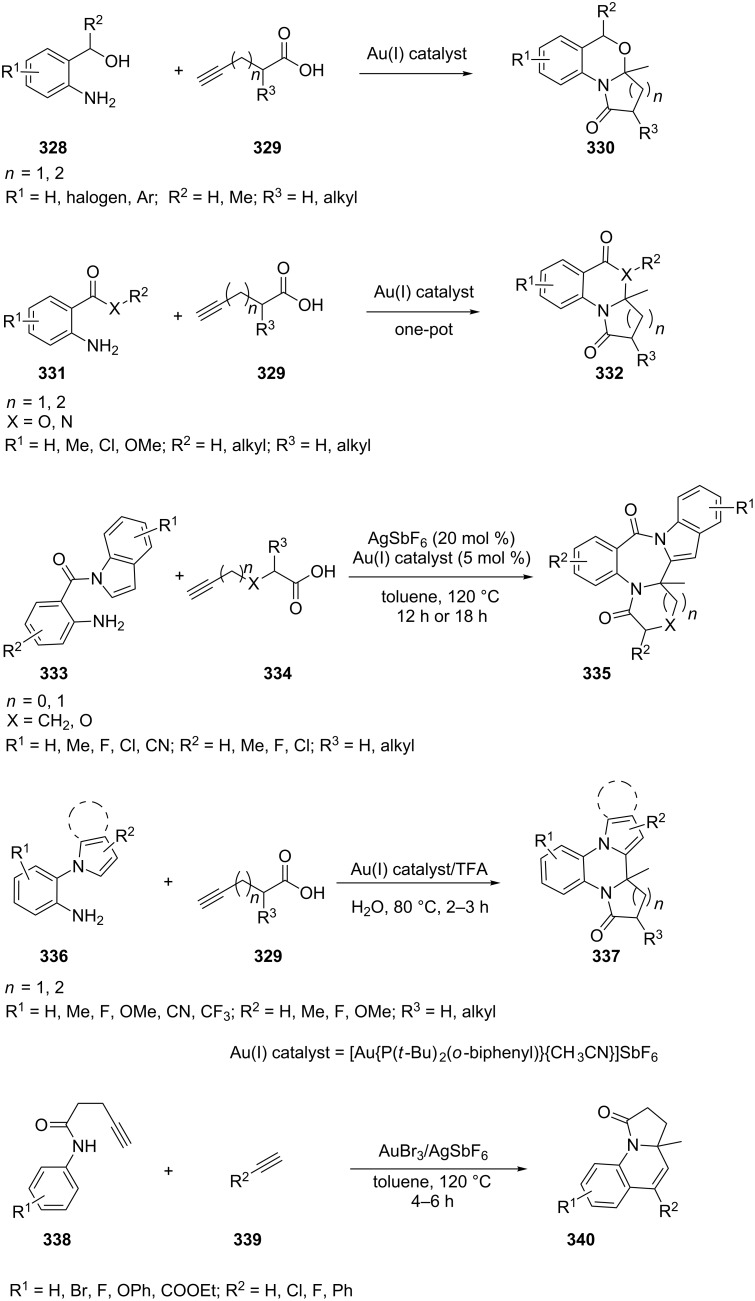
Gold-catalyzed tandem reactions of alkynes.

In an attempt to devise an efficient synthesis of potential bioactive fused heterocyles, our group developed a highly efficient, [Au{P(*t*-Bu)_2_(*o*-biphenyl)}{CH_3_CN}]SbF_6_-catalyzed cascade cycloisomerization to produce pyrrolo/pyrido[2,1-*b*]benzo[*d*][1,3]oxazin-1-ones **330** [161], pyrrolo/pyrido[2,1-*a*][1,3]benzoxazinones **332** [162], benzo[*e*]indolo[1,2-*a*]-pyrrolo[2,1-*c*][1,4]diazepine-3,9-diones **335** [163], and fused quinoxalinones **337** [164]. These cascades are proposed to occur from an initial enol lactone intermediate via an intramolecular cycloaddition [165]. A subsequent intermolecular hydroamination of the intermediate, followed by a cyclization, leads to the observed products. Our group also investigated the construction of highly functionalized pyrrolo[1,2-*a*]quinolin-1(2*H*)-ones **340** via a AuBr_3_/AgSbF_6_-catalyzed cascade transformation sequence (Scheme 56). The strategy affords a straightforward and efficient construction of tricyclic lactam molecular architectures in which several carbon–carbon and carbon–nitrogen bonds are formed in a one-pot reaction from simple starting materials [166].

**Scheme 57:**
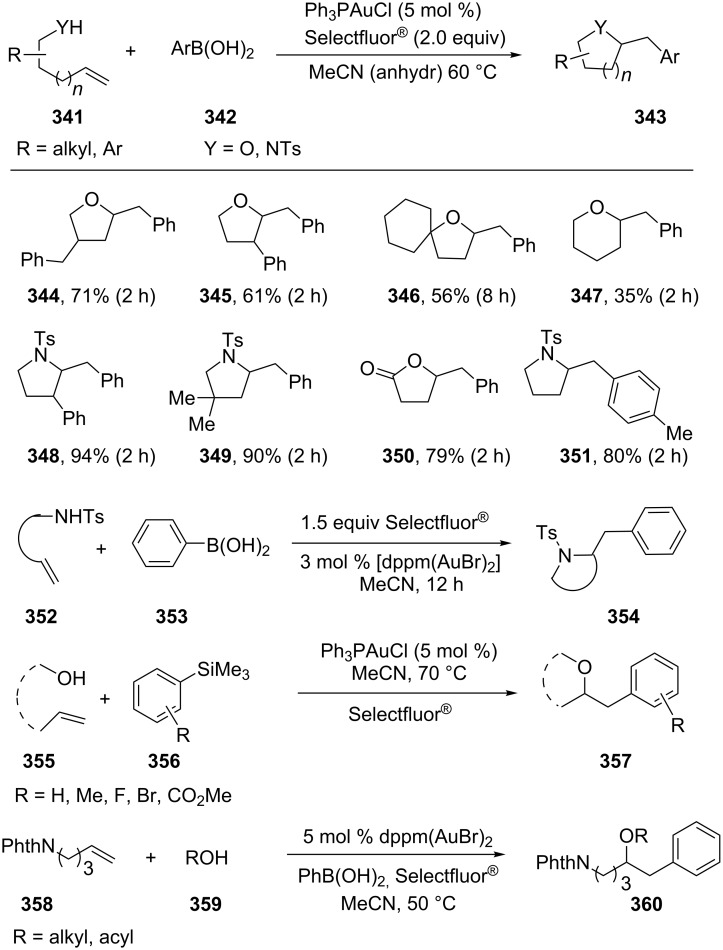
Aminoarylation and oxyarylation of alkenes.

The catalytic conversion of C(sp^3^)–Au bonds into C(sp^3^)–C(sp^2^) bonds is an ongoing challenge. In 2010, Zhang’s group reported the first example in an intermolecular oxidative cross-coupling manner [167]. In their pioneering work, carboamination, carboalkoxylation and carbolactonization of terminal alkenes **341** was achieved via oxidative gold catalysis and provided expedient access to various substituted *N*- or *O*-heterocycles (**344**–**351**) (Scheme 57). Deuterium labeling experiments were carried out to unveil the reaction mechanism. The results established the anti nature of the alkene functionalization and the indispensable role of Au(I)/Au(III) catalysis. Toste’s group and Russell’s group subsequently reported the aminoarylation and oxyarylation of alkenes (**352** and **355**) following a similar protocol [168–169]. In the gold-catalyzed intramolecular aminoarylation of alkenes, ligand and halide effects played a dramatic role for the addition to alkenes. The experimental studies suggest that the C–C bond-forming reaction occurs through a bimolecular reductive elimination. Furthermore, a gold-catalyzed three-component coupling was also developed for the oxidative oxyarylation of alkenes **358** via a similar strategy [170].

**Scheme 58:**
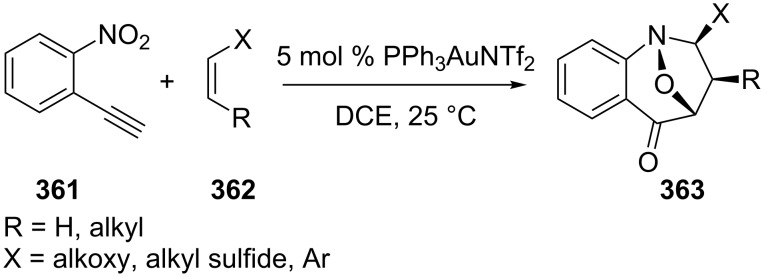
Cycloaddition of 2-ethynylnitrobenzene with various alkenes.

From the discovery and development of metal–carbenoids in cycloadditions with alkenes, as well as the internal redox reactions on alkynes, a further extensive investigation was focused on the new redox/cycloaddition cascades on alkynes to obtain azacyclic compounds **363** [171]. The central cores of the products were constructed through a formal [2 + 2 + 1] cycloaddition that involved α-carbonyl–carbenoids, nitroso species and external alkenes (Scheme 58).

**Scheme 59:**
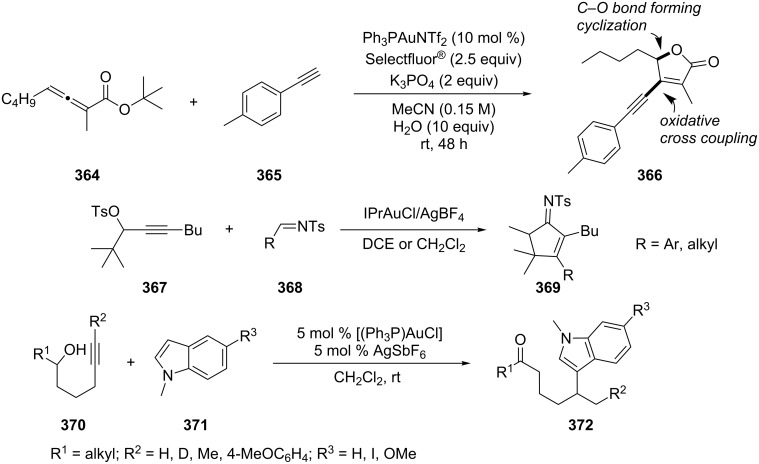
Gold-catalyzed tandem reactions of allenoates and alkynes.

A gold(I)-catalyzed cascade cyclization/oxidative cross-coupling process has been devised to prepare β-alkynyl-γ-butenolides **366** directly from allenoates **364** and various terminal alkynes [172]. The González group developed an intermolecular reaction of internal alkynes and imines, in which the propargyl tosylates **367** react with *N*-tosylaldimines **368** to afford cyclopent-2-enimines **369** [173]. The final product was achieved through a 1,2-migration of the tosylate followed by the interaction with the imine and a Nazarov-like cyclization. Barluenga et al. reported a gold-catalyzed cascade reaction involving an unusual intramolecular redox process in which 5-heteroaryl-substituted ketone derivatives **372** were obtained from secondary 5-hexyn-1-ols **370** (Scheme 59) [174]. The first step is supposed to be an intramolecular addition of the hydroxy group to the internal carbon of the triple bond, which is similar to the mechanism mentioned above [161,163].

### 6 Gold-catalyzed asymmetric addition reactions

The chiral ligand used for the transition metal-catalyzed reactions are the main determinant of enantioselectivity. Although asymmetric catalysis using chiral organometal complexes and chiral organomolecules have shown many advantages and a range of catalytic asymmetric reactions have been well documented [175], gold-catalyzed asymmetric addition reactions do not feature often. More recently this situation has been changing with significant progress being made in this area. To date, a broad range of chiral catalysts have been developed. Despite the large amount of chiral ligands used, only a few provided good to high enantioselectivities. The best ee values have been obtained with thiourea-cinchonine [176], chiral carbene [177], BINAP [178–180], and BIPHEP [181–190] analogs.

**Scheme 60:**
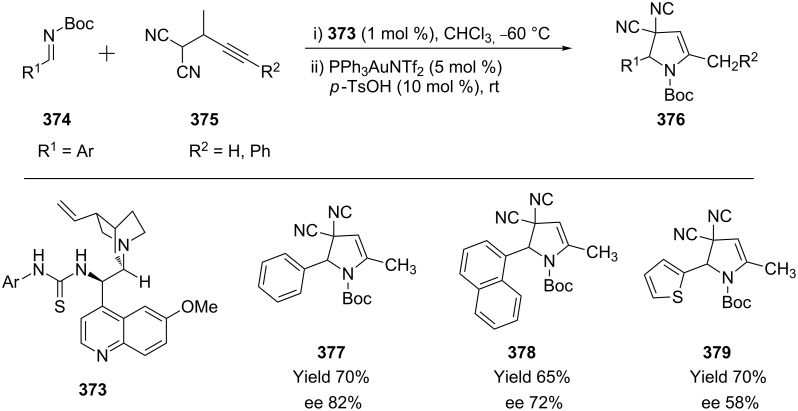
Gold-catalyzed asymmetric synthesis of 2,3-dihydropyrroles.

Monge et al. reported a direct asymmetric one-pot synthesis of optically active 2,3-dihydropyrroles from propargyl malononitriles **375** and *N*-Boc-protected imines **374** (Scheme 60) [176]. In the alkyne hydroamination (which is based on a bifunctional organocatalytic Mannich-type reaction, subsequent gold-catalyzed alkyne hydroamination and isomerization) thiourea-based hydrogen bonding organocatalyst **373** and PPh_3_AuNTf_2_ proved to be compatible upon protonation with *p*-TsOH. Electron-poor aromatic imines can be employed to give the corresponding 2,3-dihydropyrroles **376** in good yields (74–80%) and enantioselectivities (68–72% ee). However, lower enantioselectivity may result from the more electron-rich substituent groups. For example, the heteroaromatic thiophene-based imine gave the desired products **379** in good yield (70%), albeit in moderate enantioselectivity (58% ee).

**Scheme 61:**
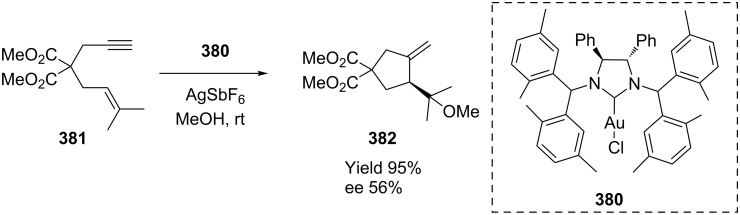
Chiral [NHC–Au(I)]-catalyzed cyclization of enyne.

In the study of enantioselective cyclization, for example, of 1,6-enynes **381** for the synthesis of cyclopentane derivatives **382**, Matsumoto and co-workers found chiral carbene–AuCl catalyst precursor **380** gave moderate enantioselectivity of up to 59% (Scheme 61) [177].

**Scheme 62:**
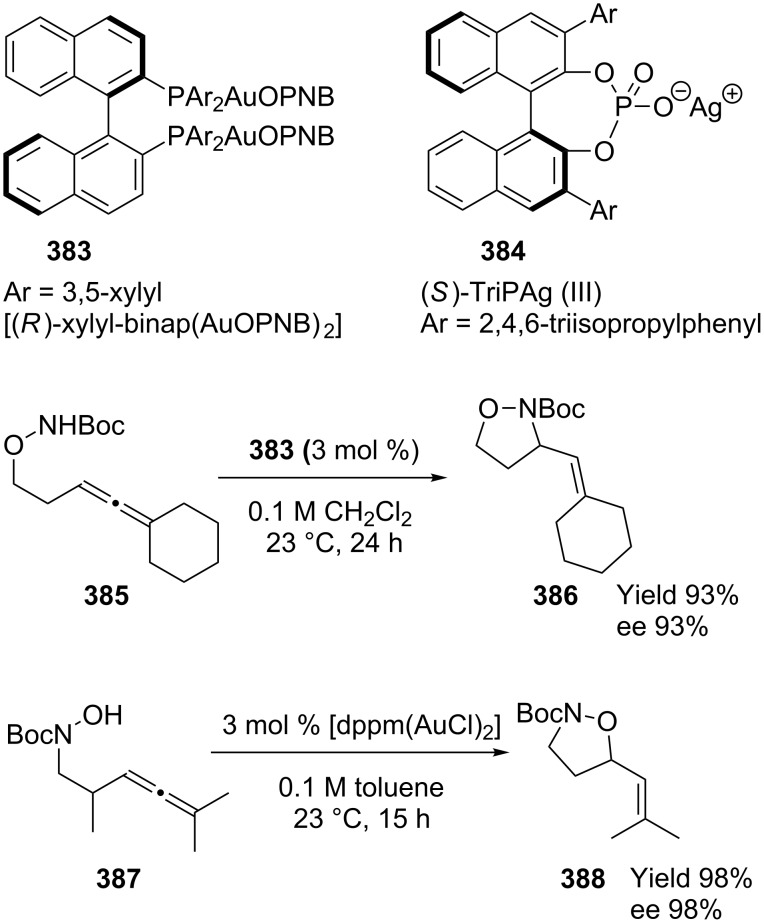
Gold-catalyzed hydroaminations and hydroalkoxylations.

In the last 3 years, enantioselective gold-catalyzed reactions with BINAP and BIPHEP analogs have been far more documented compared to other ligands. In 2009, Toste’s group reported the application of [(*R*)-xylyl-binap-(AuOPNB)_2_] **383** in gold-catalyzed hydroaminations and hydroalkoxylations of allenes with hydroxylamines and hydrazines, which gave ee values of up to 99% [178]. Whereas chiral biarylphosphinegold(I) complexes are suitable catalysts for the enantioselective addition of nitrogen nucleophiles to allenes, the addition of oxygen nucleophiles requires the use of chiral anions **384** (Scheme 62).

**Scheme 63:**
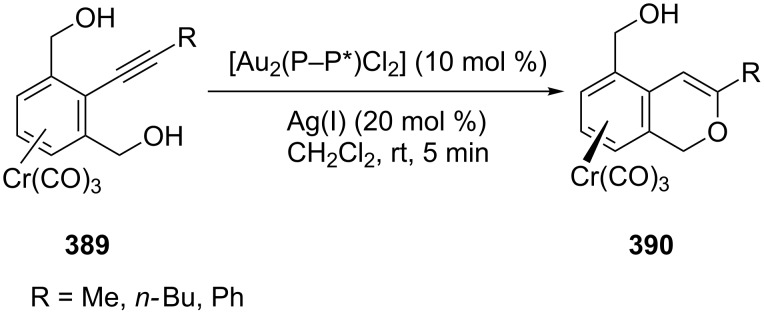
Gold(I)-catalyzed asymmetric hydroalkoxylation of 1,3-dihydroxymethyl-2-alkynylbenzene chromium complexes.

Gold(I)-catalyzed asymmetric cyclization of 1,3-dihydroxymethyl-2-alkynylbenzene chromium complexes **389** gave planar chiral isochromene–chromium complexes **390** with high enantioselectivity [179]. Enantioselectivities of the cyclized isochromene–chromium complexes are largely dependent on the combination of gold pre-catalysts and silver salts. The use of AgSbF_6_ resulted in excellent enantioselectivities, regardless of the nature of the gold pre-catalyst (Scheme 63).

**Scheme 64:**
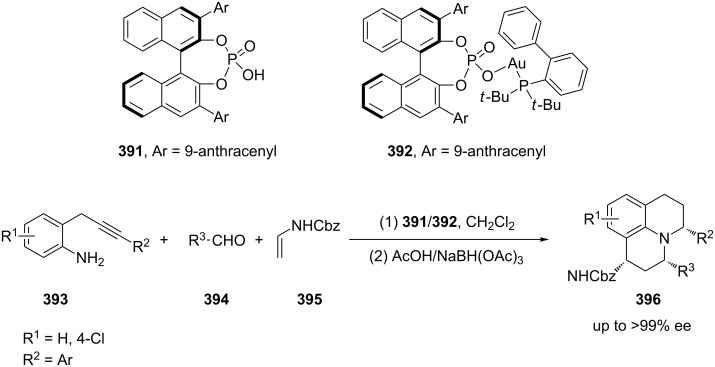
Gold-catalyzed synthesis of julolidine derivatives.

Julolidine derivatives **396** were obtained via a highly enantioselective three-component (**393**–**395**) cascade reaction which involved an enantioselective [4 + 2] cycloaddition reaction catalyzed by a chiral phosphoric acid and a subsequent catalytic intramolecular hydroamination by a gold(I) complex (Scheme 64) [180]. Further studies revealed that the Brønsted acid is both a chiral catalyst for the asymmetric cycloaddition and assists to facilitate the gold complex catalyzed hydroamination.

**Scheme 65:**
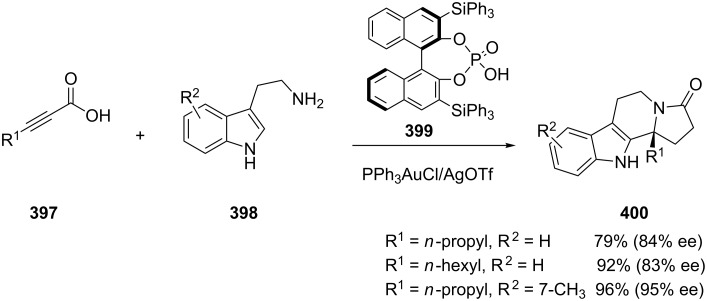
Gold-catalyzed the synthesis of chiral fused heterocycles.

Muratore et al. have reported an interesting example of C–N bond formation for the construction of chiral nitrogen-containing fused heterocycles **400** [191]. In this case, different alkynoic acids **397** were treated with Ph_3_PAuCl/AgOTf and tryptamines **398** in the presence of (*R*)-3,3'-bis(triphenylsilyl)BPA **399**. The multi-catalyst cascade products were isolated in good yields and with high ee values (Scheme 65).

**Scheme 66:**
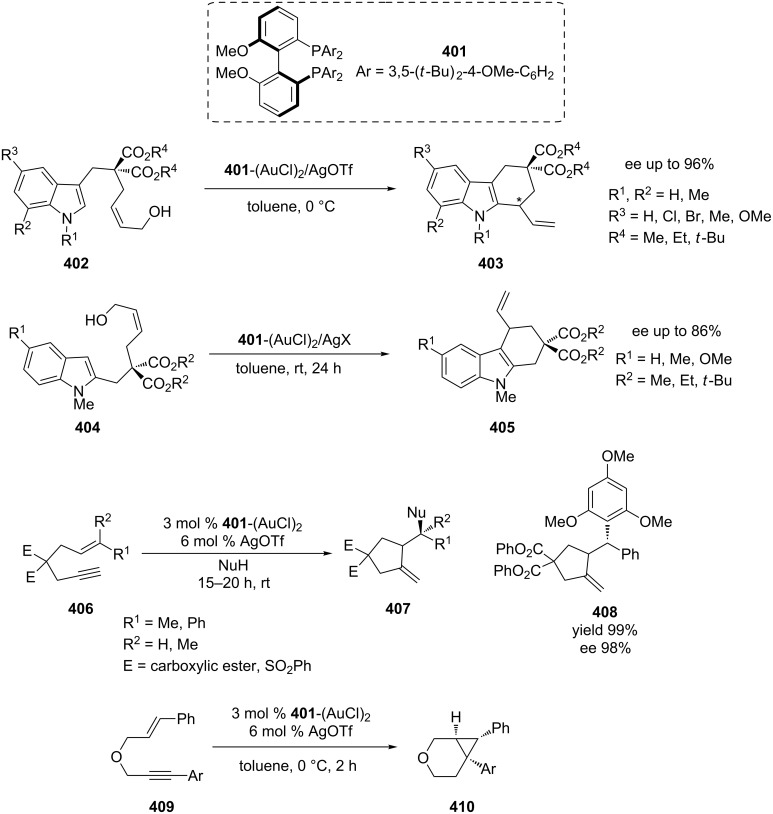
Gold-catalyzed asymmetric reactions with 3,5-(*t*-Bu)_2_-4-MeO-MeOBIPHEP.

BIPHEP is the most extensively used chiral atropisomeric biaryl diphosphine ligand in the gold catalytic enantioselective addition. Although the gold catalysis has been well developed, the use of non-activated olefinic C–C double bonds is still largely unexplored due to the intrinsic inertness of C=C (with respect to allenes and alkynes) in taking part in nucleophilic addition reactions assisted by π-electrophilic activation [183]. The first example of a direct catalytic enantioselective Friedel–Crafts allylic alkylation reaction with alcohols was reported by Bandini’s group [182]. In terms of stereo-induction, 3,5-(*t*-Bu)_2_-4-MeO-MeOBIPHEP **401** (Scheme 66) gave the best results. Their method exploits the unprecedented capability of chiral gold(I) catalysts to activate selectively prochiral π-activated alcohols **402** toward aromatic functionalization in a highly enantioselective manner. On the basis of the above results, the same group extended the substrate scope of the 3,5-(*t*-Bu)_2_-4-MeO-MeOBIPHEP–Au-catalyzed Friedel–Crafts-type alkylation to indolyl alcohols **404** bearing an unsaturated side chain at the C2 position of the indole [183]. 1,6-Enyne derivatives and their analogs are the most frequently used substrates for gold-catalyzed cycloisomerization. Chao et al. discovered that the combination of atropisomeric electron-rich and hindered chiral ligand 3,5-(*t*-Bu)_2_-4-MeO-MeOBIPHEP **401** with Au(I) and silver salts promoted the enantioselective hydroarylation/cyclization reaction of 1,6-enynes **406** under mild conditions [181]. Treatment of enynes with catalytic amount of 3,5-(*t*-Bu)_2_-4-MeO-MeOBIPHEP(AuCl)_2_ and AgOTf in Et_2_O at room temperature for 15–20 hours led to the desired arylated products with ee values up to 98%. A similar strategy was also applied by the same group in the asymmetric Au(I)-catalyzed synthesis of bicyclo[4.1.0]heptene derivatives **410** via a cycloisomerization process of 1,6-enynes **409** [184].

**Scheme 67:**
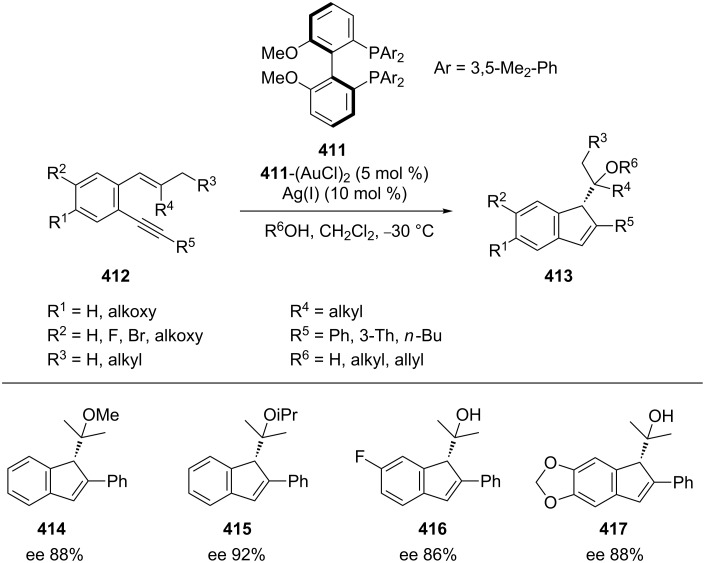
Gold-catalyzed cyclization of *o*-(alkynyl) styrenes.

Employing the atropisomeric electron-rich ligand 3,5-xylyl-MeOBIPHEP **411** (Scheme 67), Sanz’s group has developed an asymmetric gold-catalyzed cycloisomerization or alkoxycyclization of *o*-alkynylstyrenes **412** to prepare enantiomerically enriched functionalized 1*H*-indene derivatives **413** (including **414**–**417**) with high ee values (up to 92%) [190].

**Scheme 68:**
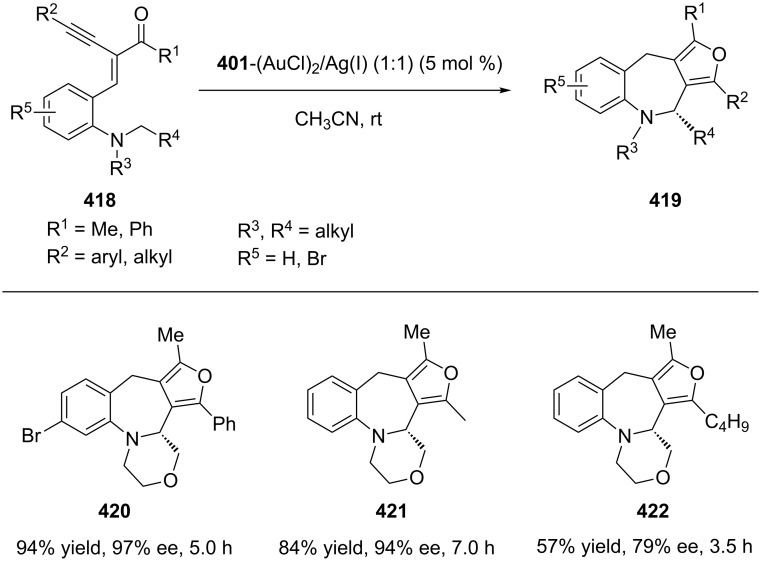
Asymmetric gold(I)-catalyzed redox-neutral domino reactions of enynes.

Due to the strength of sp^3^ C–H bonds and because it can be difficult for the metal to reach sterically hindered C–H bonds, direct functionalization of sp^3^ C–H bonds remained a challenge for a long time. Recently, however, Zhang’s group have presented the first example of an enantioselective redox-neutral domino reaction catalyzed by gold(I) that results in the direct functionalization of unreactive sp^3^ C–H bonds. Furan-fused azepine derivatives **419** (including **420**–**422**) have been obtained from enyne **418** with high enantioselectivities (Scheme 68) [185].

**Scheme 69:**
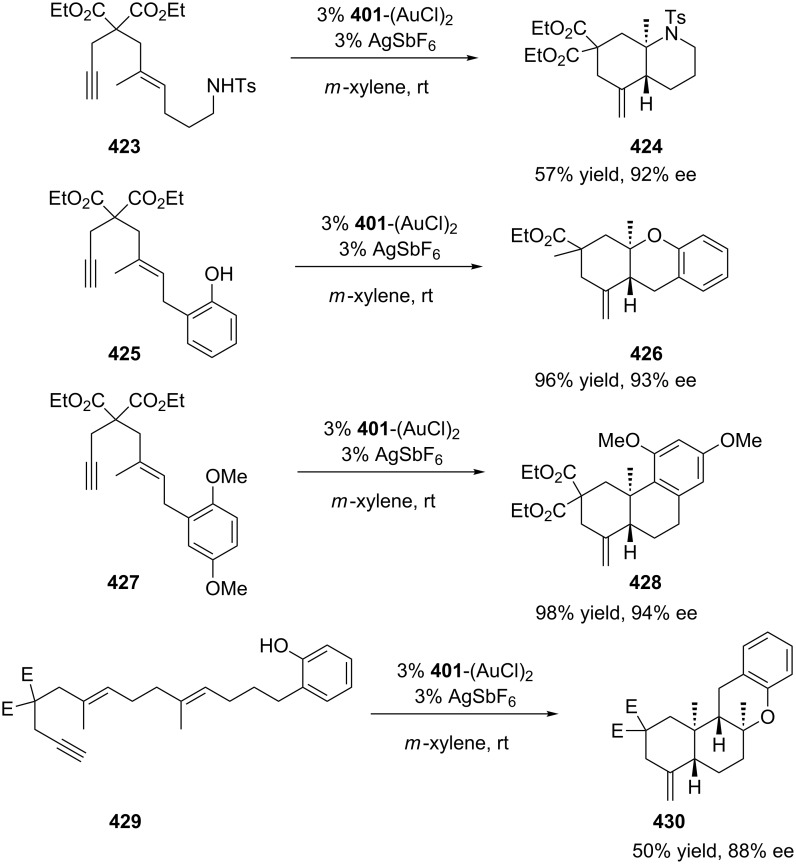
Gold(I)-catalyzed enantioselective polyene cyclization reaction.

Toste’s group developed the first example of a highly enantioselective polyene (**423**, **425**, **427**, **429**) cyclization reaction in which transition metal-promoted alkyne activation serves as the cyclization initiating event [186]. The reactions of the enyne with the monocationic gold(I) complexes and AgSbF_6_ were carried out in the presence of sterically encumbered phosphines. The use of 3,5-(*t*-Bu)_2_-4-MeO-MeOBIPHEP **401** resulted in the formation of fused bicyclic compounds (**424**, **426**, **428**, **430**) with good ee values (Scheme 69).

**Scheme 70:**
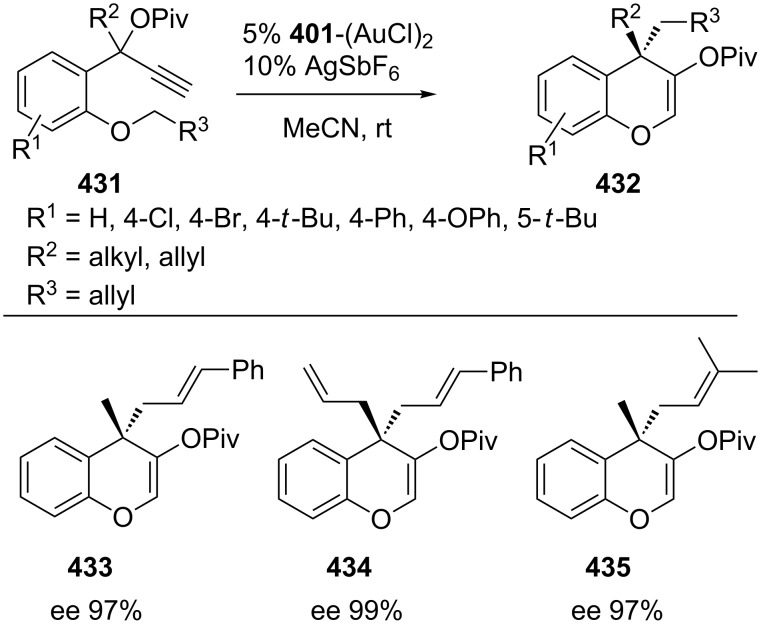
Gold(I)-catalyzed enantioselective synthesis of benzopyrans.

**Scheme 71:**
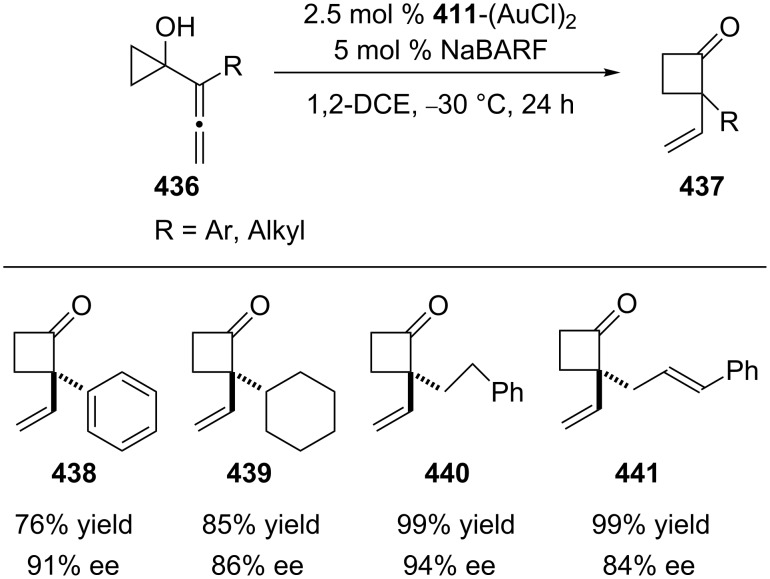
Gold(I)-catalyzed enantioselective ring expansion of allenylcyclopropanols.

The 3,5-(*t*-Bu)_2_-4-MeO-MeOBIPHEP–Au complex was also employed in the carboalkoxylation reaction of propargyl esters **431** to afford benzopyrans **432** containing quaternary stereocenters with excellent enantioselectivity (Scheme 70) [187]. Kleinbeck and Toste developed a gold(I)-catalyzed enantioselective ring expansion of allenylcyclopropanols **436** with the chiral ligand 3,5-xylyl-MeOBIPHEP **411** to obtain cyclobutanones **437** (including **438**–**441**) (Scheme 71) [188]. Notably, the amount of catalyst could be reduced without significant loss of enantioselectivity or yield.

## Conclusion

In this account, we have presented a summary of the recent gold catalysis which involves the addition of X–H (X = O, N, C) bonds to C–C multiple bonds, tandem reactions, and asymmetric additions. The variety of reactions reflects that gold catalysis has become a very innovative synthetic tool in modern organic chemistry. What is particularly worth mentioning is that the design or choice of chiral ligands together with gold catalysts is the key to attaining high asymmetric induction. Up to now, only a small proportion of the chiral ligands have been successfully introduced to gold-catalyzed reactions. Consequently, the development of new and efficient chiral ligands or chiral gold complexes is still a major challenge for the future.
